# Repurposing of approved drugs with potential to interact with SARS-CoV-2 receptor

**DOI:** 10.1016/j.bbrep.2021.100982

**Published:** 2021-03-29

**Authors:** Tamim Ahsan, Abu Ashfaqur Sajib

**Affiliations:** a Department of Genetic Engineering & Biotechnology, Bangabandhu Sheikh Mujibur Rahman Maritime University, Dhaka, 1216, Bangladesh; b Department of Genetic Engineering & Biotechnology, University of Dhaka, Dhaka, 1000, Bangladesh

**Keywords:** COVID-19, SARS-CoV-2, Spike protein, ACE1, ACE2, Host-virus interaction, Drug repurposing

## Abstract

Respiratory transmission is the primary route of Severe Acute Respiratory Syndrome Coronavirus 2 (SARS-CoV-2) infection. Angiotensin I converting enzyme 2 (ACE2) is the known receptor of SARS-CoV-2 surface spike glycoprotein for entry into human cells. A recent study reported absent to low expression of ACE2 in a variety of human lung epithelial cell samples. Three bioprojects (PRJEB4337, PRJNA270632 and PRJNA280600) invariably found abundant expression of ACE1 (a homolog of ACE2 and also known as ACE) in human lungs compared to very low expression of ACE2. In fact, ACE1 has a wider and more abundant tissue distribution compared to ACE2. Although it is not obvious from the primary sequence alignment of ACE1 and ACE2, comparison of X-ray crystallographic structures show striking similarities in the regions of the peptidase domains (PD) of these proteins, which is known (for ACE2) to interact with the receptor binding domain (RBD) of the SARS-CoV-2 spike protein. Critical amino acids in ACE2 that mediate interaction with the viral spike protein are present and organized in the same order in the PD of ACE1. *In silico* analysis predicts comparable interaction of SARS-CoV-2 spike protein with ACE1 and ACE2. In addition, this study predicts from a list of 1263 already approved drugs that may interact with ACE2 and/or ACE1 and potentially interfere with the entry of SARS-CoV-2 inside the host cells.

## Highlights

• Peptidase domains (PD) of ACE1 and ACE2 have striking similarities.
• *In silico* analysis predicts comparable interactions of S protein with ACE1 and ACE2.
• Several approved drugs may be repurposed to interfere with SARS-CoV-2 binding.

## Graphical abstract

**Figure:**
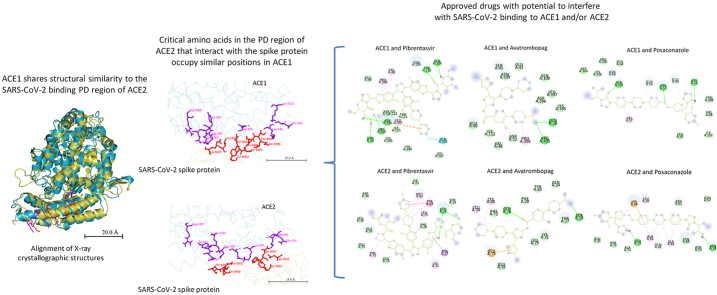

## 1 Introduction

Coronavirus disease (COVID-19) is an acute infectious disease caused by the Severe Acute Respiratory Syndrome Coronavirus 2 (SARS-CoV-2) [1]. Corona viruses are enveloped viruses with a positive-sense single-stranded ribonucleic acid (RNA) genome [2]. Respiratory transmission is the primary route of SARS-CoV-2 infection [3,4], which shares a similar mechanism with SARS-CoV (caused an outbreak in 2003) in making its way inside the host cells [5,6]. Angiotensin I converting enzyme 2 (ACE2) is the known cellular receptor for both SARS-CoV and SARS-CoV-2 in human [6,7]. The receptor binding domain (RBD) of the surface spike glycoprotein (S protein) of these viruses interact with the extracellular peptidase domain (PD) of ACE2 using electrostatic as well as van der Waals (vdW) forces [6,[8], [9], [10]]. Despite their overall similarities in structures, SARS-CoV-2 spike protein has evolved with a number of sequence variations and conformational deviations from that of SARS-CoV in the RBD that interact with ACE2 [[6], [7], [8],^11^]. Structural analyses have revealed the key interactions between the SARS-CoV-2 spike protein RBD and ACE2 [[6], [7], [8],^11^]. With its modified spike protein SARS-CoV-2 is assumed to bind human ACE2 more efficiently than SARS-CoV [7,8,11]. Binding affinity of the surface spike protein to ACE2 is one of the most important determinants of SARS-CoV-2 infectivity [7]. SARS-CoV-2 might have gained its tremendous capability to infect and transmit in humans through enhanced binding to host receptor.

ACE2 plays an important role in the maturation of angiotensin, which controls vasoconstriction and blood pressure [12]. ACE2 is a homolog of angiotensin converting enzyme (ACE1/ACE) with subtle differences in the active site [13,14]. Whereas ACE2 acts as a carboxypeptidase that removes a single amino acid from the C-terminus of susceptible substrates, ACE1 acts as a carboxy-dipeptidase (or, peptidyl-dipeptidase) and removes a C-terminal dipeptide [15]. A recent study reported absent to low level of ACE2 expression in a variety of human lung epithelial cell samples and suggested for alternative receptors that may facilitate SARS-CoV-2 mediated host cell infection [16]. Three bioprojects (PRJEB4337, PRJNA270632 and PRJNA280600) invariably found very low expression of ACE2 in human lungs, whereas ACE1 was found to be more abundantly expressed. Besides, *ACE1* (I/D) polymorphism may be a predictor of the clinical outcomes of COVID-19 and explain interpopulation differences in COVID-19 severity [17,18]. Till June 30, 2020 COVID-19 has spread in 216 countries and regions on earth with over 10,185,000 confirmed cases of infection and more than 503,500 deaths (WHO Coronavirus disease (COVID-19) Situation Report-162). Despite an urgent need to find options to help tens of thousands of patients and preclude potential death, there is no decidedly proven therapy to treat COVID-19 [1,19]. Repurposing of already approved drugs, if available, may be an immediate and promising option to tackle COVID-19. One strategy might be the use of a drug that binds to the site that is recognized by the RBD of SARS-CoV-2 surface spike protein, and thus interfere with its entry into the host cells.

This *in silico* study explored the possibility of SARS-CoV-2 spike protein interaction with ACE1, which is more abundant than ACE2 in human lungs as well as other organs. This study also explored the prospect of repurposing already approved drugs that may interact with ACE2 and/or ACE1 to potentially interfere with the entry of SARS-CoV-2 inside the host cells.

## 2 Materials and methods

### 2.1 Comparison of X-ray crystallographic structures of ACE1 and ACE2

X-ray crystallographic structures of human ACE1 (PDB ID:1O86) [20], ACE2 (PDB ID: 6LZG) [21] and SARS-CoV-2 spike protein (PDB ID: 6VYB) [22] were retrieved from the Research Collaboratory for Structural Bioinformatics (RCSB) Protein Data Bank (PDB) [23]. These structures were processed (*i.e.* removal of hetero atoms/HETATM, inhibitor and monomerization) using Discovery Studio Visualizer (v20.1.0.19295) [24]. 3D structures were aligned using RaptorX alignment tool [25]. Aligned 3D models were analyzed using CCP4mg [26].

### 2.2 Prediction of interaction between ACE1 and SARS-CoV-2 surface spike glycoprotein

Interaction of ACE1 and ACE2 with SARS-CoV-2 surface spike glycoprotein were predicted using HADDOCK2.2 tool [27]. Predicted protein complexes were analyzed using PyMOL [28], CCP4mg [26] and Discovery Studio Visualizer (v20.1.0.19295) [24].

### 2.3 *In silico* assessment of drugs with potential to block SARS-CoV-2 spike protein interaction with ACE1 and ACE2

Twelve hundred and sixty three approved drugs (Supplementary Table 2) in 3D SDF format were retrieved from DrugBank [29], BindingDB [30], e-Drug3D [31] databases. Interaction of these drugs with ACE1 and ACE2 were predicted using AutoDock Vina in PyRx [32,33]. These structures were further analyzed using CCP4mg [26].

## 3 Results

### 3.1 Alignment of ACE1 and ACE2 X-ray crystallographic structures

**Fig. 1:**
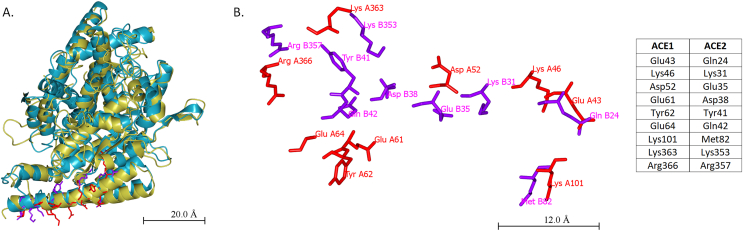
Alignment of X-ray crystallographic structures of ACE1 (PDB ID:1O86) and ACE2 (PDB ID: 6LZG). A. SARS-CoV-2 spike protein binding region (RBD) of ACE1 (in dark cyan) and ACE2 (in gold) have similar tertiary structures in the PD region. B. Glu43, Lys46, Asp52, Glu61, Tyr62, Glu64, Lys101, Lys363 and Arg366 in ACE1 (in red) are positioned in similar order to Gln24, Lys31, Glu35, Asp38, Tyr41, Gln42, Met82, Lys353 and Arg357 in ACE2 (in purple). Chain A and B represent ACE1 and ACE2, respectively.

Alignment of X-ray crystallographic structures of ACE1 and ACE2 reveals striking similarities in the tertiary structures of their peptidase domains (Fig. 1A). Peptidase domain of ACE2 is known to interact with the RBD of SARS-CoV-2 spike protein. Amino acid residues in this region of ACE2 (Gln24, Lys31, Glu35, Asp38, Tyr41, Gln42, Met82, Lys353, Arg357) that interact with the spike protein [4,6] are also present (or, amino acids with similar polarity and structures) in the peptidase domain of ACE1 (Fig. 1B). Although it is not obvious in the aligned primary sequences, these important amino acid residues in the PD of ACE1 and ACE2 are present in the same order in their tertiary structures (Fig. 1B). Lys353 in the PD of ACE2 is critically important in binding with the SARS-CoV-2 RBD [11]. Lys363 in the PD of ACE1 is present in a similar position (Fig. 1B).

### 3.2 Predicted interactions of SARS-CoV-2 surface spike glycoprotein with ACE1 and ACE2

**Fig. 2:**
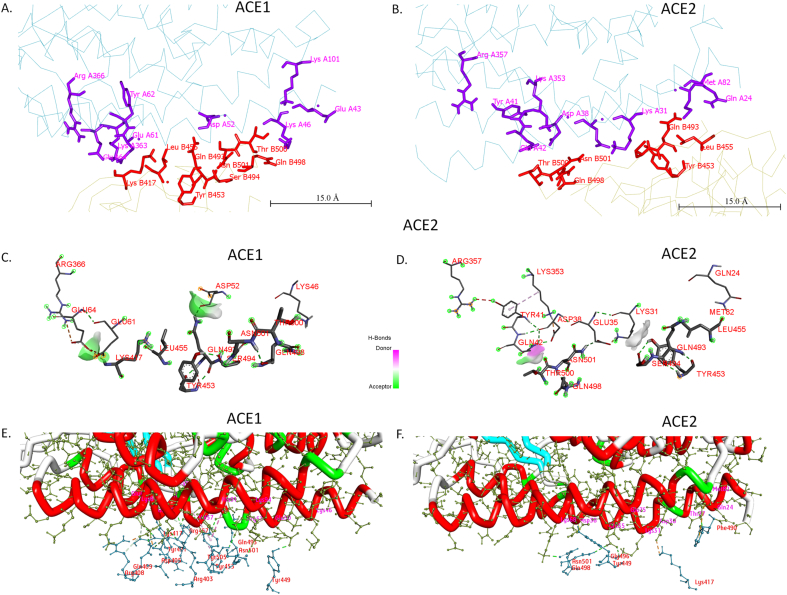
Predicted interactions of ACE1 and ACE2 with the RBD of SARS-CoV-2 surface spike protein. A and B. Amino acid residues at the interface of ACE1 and ACE2 PD regions (in purple) with the RBD of SARS-CoV-2 spike protein (in red). Chain A and B represent ACE1/ACE2 and spike protein, respectively. C and D. Specific interactions of comparable amino acids at the ACE1 and ACE2 PD regions with the RBD of SARS-CoV-2 spike protein. E and F. All interactions at the ACE1 and ACE2 PD regions with the RBD of SARS-CoV-2 spike protein.

Receptor-ligand interaction analysis using molecular docking technique could predict the amino acids at the interface of ACE1 and ACE2 peptidase domains with the RBD of the spike protein (Fig. 2). Although amino acid residues at the interface of ACE2 and spike proteins are already known from X-ray crystallographic analysis, this *in silico* prediction was performed as a control to assess the performance of the docking process. This also allowed the direct comparison between the interacting sites of ACE1 and ACE2 with the RBD of SARS-CoV-2 spike protein based on a common platform. The amino acid residues of ACE2 at the interface with the SARS-CoV-2 spike protein matched to the previous reports [[6], [7], [8],^11^]. Similar and more residues were observed in the predicted interactions between ACE1 and the spike protein (Supplementary Table 1). Earlier studies have reported predominantly electrostatic interactions along with van der Waals forces between ACE2 and the RBD of spike protein [6,8]. The predicted interactions of ACE1 and ACE2 with the spike protein involve similar forces (Table 1).

**Table 1:** Predicted interactions of ACE1 and ACE2 with the RBD of SARS-CoV-2 spike protein.

**Feature**	**ACE1 and spike protein**	**ACE2 and spike protein**
Van der Waals energy (kcal/mol)	−48.8 ± 3.3	−59.6 ± 4.7
Electrostatic energy (kcal/mol)	−319.7 ± 36.8	−122.1 ± 46.9
Desolvation energy (kcal/mol)	87.4 ± 7.4	33.8 ± 14.9
Z-Score	−1.2	−1.4
RMSD from the overall lowest-energy structure	1.7 ± 0.3	1.1 ± 0.7

### 3.3 Drugs with potential to block SARS-CoV-2 spike protein interaction with ACE1 and ACE2

**Fig. 3:**
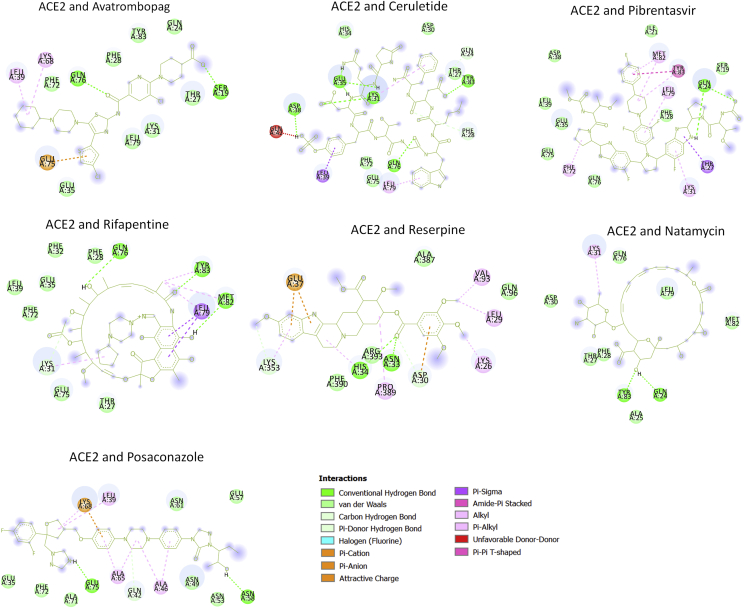
Drugs with potential to block SARS-CoV-2 surface glycoprotein interaction with ACE2. Interacting amino acid residues in ACE2 are shown as spheres. 2D ligand-protein diagrams were generated using Discovery Studio Visualizer based on the interactions predicted with AutoDock Vina in PyRx.

**Fig. 4:**
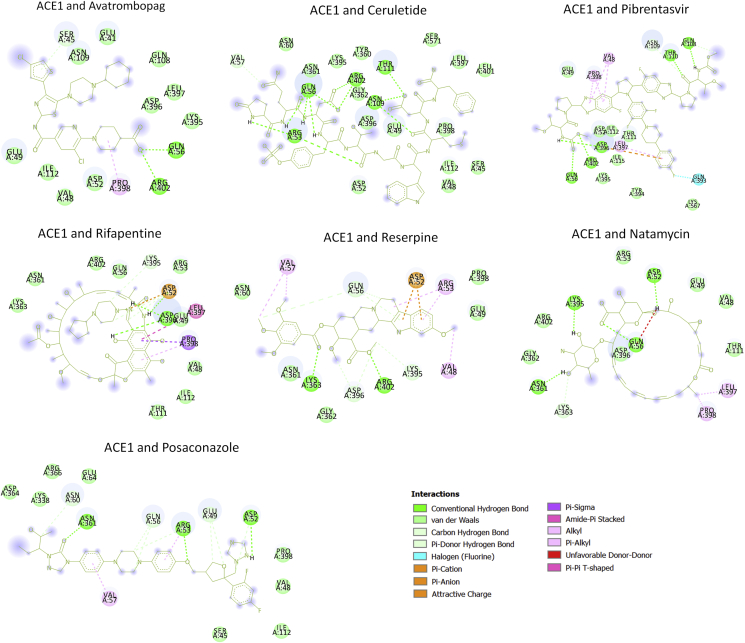
Drugs with potential to block SARS-CoV-2 surface glycoprotein interaction with ACE1. Interacting amino acid residues in ACE1 are shown as spheres. 2D ligand-protein diagrams were generated using Discovery Studio Visualizer based on the interactions predicted with AutoDock Vina in PyRx.

A total of 1263 approved drugs (Supplementary Table 2) were assessed for potential interaction with ACE1 and ACE2 at regions that overlap with the predicted and already known binding sites for the RBD of the SARS-CoV-2 spike protein, respectively. Angiotensin II is a natural substrate of ACE2 [15]. Molecular docking with AutoDock Vina predicted interaction of angiotensin II with the peptidase domain of ACE2 with a binding energy of −6.0 kcal/mol. Drugs that bind to overlapping regions in the peptidase domains of ACE1 and/or ACE2 and, therefore, may perturb interaction with the SARS-CoV-2 spike protein, and has more stable binding than the native substrate (*i.e.,* predicted to release energy > 6.0 kcal/mol) and may provide additional health benefits to the COVID-19 patients by alleviating symptoms are listed in Table 2. Table 2 also provides brief description of the drugs along with their current approval status. Some drugs have multiple statuses as these have been approved for certain condition(s), but are currently on clinical trials for one or more different indications. The listed drugs (Table 2) belong to diverse categories such as antiviral, antibacterial, antifungal, antihypertensive, anticoagulant, angiotensin II analog, immunosuppressant, antiallergic and antidiarrheal, among others. Seven of these drugs (Avatrombopag, ceruletide, natamycin, pibrentasvir, posaconazole, reserpine, and rifapentine) appear to bind to SARS-CoV-2 interacting sites in the PD regions of both ACE1 and ACE2. These predicted interactions are shown in Fig. 3, Fig. 4.

**Table 2:** List of drugs that bind to ACE and ACE2 PD regions and has more stable binding than angiotensin II (*i.e.,* predicted to release energy > 6.0 kcal/mol).

**Drug**	**Binding energy (kcal/mol)**		**Status** [29]	**Category of drug**	**Description** [29,44]
	Human ACE1	Human ACE2			
AVATROMBOPAG	−6.9	−7.4	Approved, Investigational	Anti-thrombocytopenic	A small-molecule thrombopoietin receptor agonist which increases platelet number, but does not cause platelet activation.
CERULETIDE	−6.2	−6.2	Approved	Others	Exerts stimulatory effects on the gastric, biliary, and pancreatic secretion, as well as on certain smooth muscles.
NATAMYCIN	−7.4	−6.2	Approved	Antifungal	It is used for a variety of fungal infections, mainly topically.
PIBRENTASVIR	−7.5	−6.6	Approved, Investigational	Antiviral	A direct acting antiviral agent and Hepatitis C virus (HCV) NS5A inhibitor that targets viral RNA replication and viron assembly.
POSACONAZOLE	−7.8	−6.2	Approved, Investigational	Antifungal	An antifungal drug that is used to treat invasive infections by Candida species and Aspergillus species in severely immunocompromised patients.
RESERPINE	−6.3	−6.4	Approved, Investigational	Antihypertensive	Used as an antihypertensive and an antipsychotic drug.
RIFAPENTINE	−6.4	−6.5	Approved, Investigational	Antibiotic	An antibiotic drug used in the treatment of tuberculosis.
AMPHOTERICIN B	−7.1	_	Approved, Investigational	Antifungal	Used to treat potentially life threatening fungal infections.
ANIDULAFUNGIN	−6.6	_	Approved, Investigational	Antifungal	An antifungal drug used in the treatment of the following fungal infections: Candidemia and other forms of Candida infections (intra-abdominal abscess, and peritonitis), Aspergillus infections, and esophageal candidiasis. Also considered as an alternative treatment for oropharyngealcanaidiasis.
AZITHROMYCIN	−6.6	_	Approved	Antibiotic	A broad-spectrum macrolide antibiotic with a long half-life, which is primarily used for the treatment of respiratory, enteric and genitourinary infections.
DESLANOSIDE	−7.5	_	Approved	Others	A cardiotonic glycoside used for the treatment and management of congestive cardiac insufficiency, arrhythmias and heart failure.
DIGOXIN	−7.7	_	Approved	Others	A commonly used agent to manage atrial fibrillation and the symptoms of heart failure.
EPTIFIBATIDE	−7.4	_	Approved, Investigational	Anticoagulant	A synthetic cyclic hexapeptide that inhibits platelet aggregation.
ICATIBANT	−7.3	_	Approved, Investigational	others	A synthetic peptidomimetic drug that is used in acute attacks of hereditary angioedema.
NYSTATIN	−6.8	_	Approved	Antifungal	An antifungal drug that has broad-spectrum fungicidal and fungistatic activity against a number of yeasts and fungi, most notably Candida species.
RIFAMYCIN	−6.3	_	Approved, Investigational	Antidiarrheal	It is indicated for the treatment of adult patients with travelers' diarrhea caused by noninvasive strains of *E. coli*.
RIFAXIMIN	−6.6	_	Approved, Investigational	Antidiarrheal	A semisynthetic, rifamycin-based non-systemic antibiotic used in treatment of traveller's diarrhea caused by *E. coli,* reduction in risk of overt hepatic encephalopathy recurrence as well as diarrhea-predominant irritable bowel syndrome (IBS-D) in adults.
SIROLIMUS	−7.5	_	Approved, Investigational	Immunosuppressant	A potent immunosuppressant and possesses both antifungal and antineoplastic properties.
VANCOMYCIN	−7.7	_	Approved	Antibiotic	An antibacterial compound that inhibits bacterial cell wall assembly.
ALATROFLOXACIN	_	−6.4	Approved, Withdrawn	Antibiotic	It is a fluoroquinolone antibiotic.
AZILSARTAN KAMEDOXOMIL	_	−6.4	Approved, Investigational	Antihypertensive	An angiotensin II receptor antagonist indicated for the treatment of mild to moderate essential hypertension.
BALOXAVIR MARBOXIL	_	−6.4	Approved, Investigational	Antiviral	An antiviral drug for the treatment of influenza A and influenza B infections.
BETRIXABAN	_	−6.5	Approved, Investigational	Anticoagulant	A non-vitamin K oral anticoagulant whose action is driven by the competitive and reversible inhibition of the factor Xa.
BUTENAFINE	_	−6.1	Approved	Antifungal	A synthetic benzylamine antifungal agent.
CANDICIDIN	_	−6.3	Approved, Withdrawn	Antifungal	An antibiotic active against some fungi of the genus Candida.
CEFOPERAZONE	_	−6.5	Approved, Investigational	Antibiotic	A semisynthetic broad-spectrum third-generation antiobiotic effective against Pseudomonas infections. It is used in the treatment of various bacterial infections, including respiratory tract infections, peritonitis, skin infections, endometritis, and bacterial septicemia.
CELECOXIB	_	−6.5	Approved, Investigational	Antiinflammatory	A selective nonsteroidal antiinflammatory drug (NSAID) which is known for its decreased risk of causing gastrointestinal bleeding compared to other NSAIDS.
DESERPIDINE	_	−6.6	Approved	Antihypertensive	An antipsychotic and antihypertensive agent used for the control of high blood pressure and for the relief of psychotic behavior.
DIHYDROERGOTAMINE	_	−7.4	Approved	Antimigraine	A vasoconstrictor, specifically for the therapy of migraine disorders.
DORAVIRINE	_	−6.5	Approved	Antiviral	An HIV-1 non-nucleoside reverse transcriptase inhibitor (NNRTI) intended to be administered in combination with other antiretroviral medicines.
INDINAVIR	_	−7.1	Approved	Antiviral	A potent and specific HIV protease inhibitor that appears to have good oral bioavailability.
LOPERAMIDE	_	−6.3	Approved	Antidiarrheal	Long-acting synthetic antidiarrheals, which has no effect on the adrenergic system or central nervous system, but may antagonize histamine and interfere with acetylcholine release locally.
LORATADINE	_	−6.3	Approved, Investigational	Antihistamine	A second generation antihistamine used to manage symptoms of allergic rhinitis.
LUSUTROMBOPAG	_	−6.5	Approved, Investigational	Anti-thrombocytopenic	An orally bioavailable thrombopoietin receptor (TPOR) agonist, which is indicated for the treatment of thrombocytopenia in adults with chronic liver disease
MARAVIROC	_	−6.3	Approved, Investigational	Antiviral	A chemokine receptor antagonist drug that is designed to act against HIV by interfering with the interaction between HIV and CCR5
MEFLOQUINE	_	−6.1	Approved, Investigational	Antimalarial	A phospholipid-interacting antimalarial drug.
NELFINAVIR	_	−6.2	Approved	Antiviral	A potent HIV-1 protease inhibitor.
PITAVASTATIN	_	−6.1	Approved	Statin	A lipid-lowering drug belonging to the statin class of medications.
SARALASIN	_	−7.1	Investigational	Angiotensin II analog	An octapeptide analog of angiotensin II.
SIMVASTATIN	_	−6.4	Approved	Statin	Used to lower the risk of cardiovascular disease and manage abnormal lipid levels by inhibiting the endogenous production of cholesterol in the liver.
ZAFIRLUKAST	_	−7.1	Approved, Investigational	Antiasthmatic	Used for the treatment of asthma, often used in conjunction with an inhaled steroid and/or long-acting bronchodilator.

In addition to those listed in Table 2, there are other antiviral drugs (Supplementary Table 3) with potential binding abilities to ACE1 and/or ACE2. Except for baloxavir marboxil, indinavir, maraviroc, nelfinavir and pibrentasvir, the other antiviral drugs bind to sites in ACE1 and ACE2 that do not coincide with the binding of SARS-CoV-2 spike protein. A few of these antivirals are already in clinical trials as treatment options for COVID-19 [1,3,34]. Among these bictegravir, indinavir and remdesivir bind to both ACE1 and ACE2 with the release of >7 kcal/mol energy.

## 4 Discussion

### 4.1 Interaction between ACE1 and SARS-CoV-2 surface spike glycoprotein

Infection with SARS-CoV-2 affects multiple organs (including lung, liver, kidney, intestine and muscle, among others) [1,34]. Although previous studies have reported abundant expression of ACE2 on ciliated cells of the airway epithelium and alveolar type II cells in human [35], a recent study reported absent to low expression of ACE2 in human lung epithelial cells [16]. ACE1 appears to be more abundantly expressed in the COVID-19 affected organs (lung, liver, kidney, intestine and muscle) [36]. In fact, ACE1 has a wider and more abundant tissue distribution compared to ACE2 [36].

Based on the similarities to SARS-CoV spike protein, it has been suggested that SARS-CoV-2 also exploits ACE2 to mediate infection in human [7]. A number of studies have reported overlapping but different sets of amino acids in the PD of ACE2 that interact with the RBD of SARS-CoV-2. Among these Lys31 and Lys353 in ACE2 are considered as critical amino acid residues in the peptidase domain to mediate interaction with the SARS-CoV-2 spike protein [7,11]. A similar configuration of these and other important amino acid residues is present in the tertiary structure of human ACE1 enzyme (Fig. 1). Binding of SARS-CoV-2 RBD to the PD of ACE2 is driven by electrostatic interactions, which in this case is significantly stronger than the vdW interactions [8]. Alike the reported interactions between SARS-CoV/SARS-CoV-2 and ACE2 [[6], [7], [8]], the predicted interface between SARS-CoV-2 and ACE1 maintains a highly polar environment (Fig. 2 and Table 1). In fact, the predicted interaction model suggests the ACE1 and SARS-CoV-2 spike protein complex to be electrostatically more stable than the ACE2 and spike protein complex.

SARS-CoV-2 RBD has more interactions with ACE2 than the RBD of SARS-CoV, which is consistent with the higher binding affinity of SARS-CoV-2 than SARS-CoV for ACE2 [8,11]. This is attributable to the altered amino acids in the RBD of SARS-CoV-2 [8]. SARS-CoV-2 is predicted to bind ACE2 with an affinity 10 to 20 times stronger than the SARS-CoV [5,8]. As SARS-CoV-2 spike protein has evolved to bind ACE2 with higher affinity than the spike protein of SARS-CoV and gained more power to transmit and infect humans, mere speculation based on sequence comparison with SARS-CoV might not be adequate to define ACE2 as its sole receptor.

### 4.2 Repurposing of approved drugs to block SARS-CoV-2 spike protein interaction with ACE1 and ACE2

Drug repurposing is the discovery of novel therapeutic applications for already approved drugs to treat illnesses other than their primary indications [37]. This approach holds much promise as it helps to circumvent preclinical and optimization processes as well as reduce time and costs associated with drug discovery [38]. Molecular docking is one of the common computational approaches to repurpose established drugs towards novel therapeutic targets based on their structural complementarity [39]. This approach, however, has limitations particularly arising from the use of approximate scoring functions and possible imperfect binding prediction [38]. Despite these limitations, molecular docking is a well-established and experimentally validated approach for predicting drug-target associations [38]. This technique has been successfully exploited for repurposing drugs [[40], [41], [42]]. Over the last two decades, over 60 different molecular docking tools have been developed for academic and/or commercial uses. In a comparative study among these tools, AutoDock Vina, GOLD, and MOE-Dock predicted top ranking poses with the best scores [43]. AutoDock Vina applies a knowledge-based scoring function with a Monte Carlo sampling technique and the Broyden-Fletcher-Goldfarb-Shanno (BFGS) method for local optimization [32]. Their simulation results showed a significant improvement in both prediction accuracy and docking time [32,43].

In this study, drugs were docked onto ACE1 and ACE2 with AutoDock Vina [32]. Among the 1263 tested drugs, 12 appear to interact with ACE1, 22 with the ACE2 and 7 with both (with the release of >6.0 kcal/mol- the predicted binding energy of angiotensin II with ACE2) in the regions that overlap with the binding of the RBD of SARS-CoV-2 spike protein. Saralasin (an angiotensin II analog and a highly specific competitive inhibitor of angiotensin II [44] was predicted to bind at the PD of ACE2, but not ACE1, with higher affinity than angiotensin II (Table 2).

The most common symptoms of COVID-19 include fever, dry cough, breathing difficulties, chest pain, fatigue and myalgia (pain in muscles) [3]. The other less common symptoms include abdominal pain, diarrhea, nausea and vomiting [3]. COVID-19 patients also exhibit neurological symptoms such as dizziness, headache, anosmia (loss of smell), impaired consciousness, etc [1,45]. In severe cases, SARS-CoV-2 can lead to acute respiratory distress syndrome (ARDS), septic shock, metabolic acidosis, coagulation dysfunction, and eventually multiple organ failure [1,3]. No specific antiviral drugs have been confirmed to be decidedly effective against SARS-CoV-2 yet [3,34]. At present, COVID-19 patients are given supportive care and symptomatic treatments with antiinflammatory drugs and antibiotics for secondary infections [3,34].

Acute respiratory distress syndrome (ARDS) is the primary cause of death with COVID-19 [46,47]. ARDS is characterized by rapid onset of widespread inflammation in the lungs, which leads to respiratory failure. It is invoked by a “cytokine storm” [46,47] mediated by the SARS-CoV-2 stimulated systemic inflammatory response with an insurgence of pro-inflammatory cytokines (including IL-1β, IL-2,IL-6, IL7, IL-10, TNF-α, GCSF, MCP1, etc) and chemokines (CCL2, CCL3, CCL5, CXCL8, CXCL9, CXCL10, etc) [3,34]. Patients with worse outcomes and multi-organ failure (lungs, heart, kidneys and liver, among others), in particular, have significantly higher levels of IL-2, IL-6, IL-7, IL-10, GCSF, IP10, MCP1, and TNF-α [1,3,34]. Celecoxib and loratadine are two non-steroidal antiinflammatory drugs that appear to bind to the PD of ACE2 (Table 2). Sirolimus (a strong immunosuppressant), on the other hand, appears to bind to the PD of ACE1. In toxicity studies, sirolimus and loratadine have been shown to rarely cause clinically apparent liver injury [44]. These may serve as a two edged sword by blocking the binding of SARS-CoV-2 to the host receptor as well as subsiding inflammatory responses. In a mechanistic modeling approach combined with virtual screening, Loucera et al. identified sirolimus to have a strong impact over most of the specific signaling circuits in the COVID-19 [48]. Another study based on network proximity analyses of drug targets also identified sirolimus as potentially repurposable for COVID-19 [49,50].

Thrombotic complications (including thrombocytopenia, prolonged prothrombin time, and disseminated intravascular coagulation) have emerged as a critical issue in COVID-19 patients [51]. SARS-CoV-2 infection may lead to thrombocytopenia by causing destruction of platelets, reducing primary platelet production, and/or decreasing the number of circulating platelets (Xu P et al.). Avatrombopag is a small-molecule thrombopoietin receptor agonist that increases platelet number, but does not cause platelet activation [29,44]. It appears to bind at sites that overlap with the SARS-CoV-2 RBD interactions in the PD of both ACE1 and ACE2. Lusutrombopag is another antithrombocytopenic agent that binds to ACE2 in the PD region where the spike protein interacts. SARS-CoV-2-associated injury may initiate activation of coagulation and clotting cascades leading to the formation of internal blood clots [52]. Two anticoagulants eptifibatide and betrixaban dock onto the spike protein binding sites in ACE1 and ACE2, respectively. Another recent study also predicted binding of eptifibatide to the virus binding site in the ACE2 receptor [53]. Avatrombopag, lusutrombopag and betrixaban have been reported to cause unproven, but suspected rare cases of clinically apparent liver injury in toxicity assays [44].

Pibrentasvir is an antiviral drug that seems to interact with both ACE1 and ACE2 in the PD region at sites that coincide with the SARS-CoV-2 spike protein binding. Pibrentasvir is indicated for the treatment of infection mediated by Hepatitis C Virus (HCV), which is a positive-strand RNA virus [54]. Several other antiviral drugs (Baloxavir marboxil, doravirine, indinavir, maraviroc, and nelfinavir) might interact only with ACE2 in the PD region and interfere with SARS-CoV-2 binding. Except indinavir, the others (Pibrentasvir, baloxavir marboxil, doravirine, maraviroc, and nelfinavir) have been shown to cause rare cases of hepatotoxicity in toxicological studies [44]. Maffucci and Contini predicted binding of indinavir to ACE2 at the site that overlaps with the binding of SARS-CoV-2 spike protein [55]. In a virtual screening of 65 FDA approved small molecule antiviral drugs against the main protease (Mpro, also called 3CL^pro^) and the RNA-dependent RNA polymerase (RdRp), indinavir and pibrentasvir were predicted to bind Mpro of SARS-CoV-2 [56]. The same study also reported potential binding of indinavir to RdRp [56]. Several other studies also reported indinavir as a potential drug to target M^pro^/3CL^pro^ [46,47,55,57,58]. Indu et al. also reported good bioavailability of indinavir [56].

Other drugs listed in Table 2 may find purposes for other minor symptoms in COVID-19 patients. For example, loperamide and rifamycin are used as antidiarrheal drugs without evidence of liver injury in toxicity studies [44]. Secondary bacterial and/or fungal infection is an important factor affecting mortality in COVID-19 patients [49,50,59]. Although several antibacterial drugs (Alatrofloxacin, azithromycin, cefoperazone, rifapentine and vancomycin) might bind to the PD of ACE1 and/or ACE2 to obstruct SARS-CoV-2 binding, only rifapentine and vancomycin are unlikely to have any clinically apparent toxicity [44]. Vancomycin is used for treating severe infections caused by susceptible strains of methicillin-resistant (beta-lactam-resistant) *Staphylococci* [44]. It is also used to treat *Clostridium difficile* associated diarrhea and enterocolitis caused by *Staphylococcus aureus* [44]. Among the antifungal drugs (Amphotericin B, anidulafungin, butenafine, candicidin, natamycin, nystatin, and posaconazole) that bind to the PD of ACE1 and/or ACE2 with potential to affect SARS-CoV-2 binding, only anidulafungin and nystatin are unlikely to cause clinically apparent hepatotoxicity [44]. Nystatin has broad-spectrum fungicidal and fungistatic activities against a number of yeasts and fungi, most notably *Candida* species, while anidulafungin is used for the treatment of Candidemia and other forms of *Candida* infections (intra-abdominal abscess and peritonitis), *Aspergillus* infections, esophageal candidiasis and as an alternative for oropharyngeal candidiasis [29,44]. Both of these antifungal drugs appear to interact at the PD of ACE1. Posaconazole binds to the region of PDs in ACE1 and ACE2 in a manner that may impede the binding of SARS-CoV-2. Posaconazole is apparently a non-toxic drug [44]. Although treatment with posaconazole causes transient elevations in serum aminotransferase levels in 2%–12% of patients, these elevations are usually mild, asymptomatic and self-limited and rarely require discontinuation of the medication [44]. Recent studies also reported binding of nystatin and posaconazol against SARS-CoV-2 spike protein binding site [55] and M^pro^ [58]. Mohammed et al. reported potential binding of Amphotericin B to M^pro^ [58].

There are several other drugs (Table 2) that bind to the PDs of ACE1 and/or ACE2 with potential to interfere with SARS-CoV-2 binding. These include antihypertensive (Azilsartan kamedoxomil, deserpidine, and reserpine), statins (Pitavastatin and simvastatin), antimigraine (Dihydroergotamine), antiasthmatic (Zafirlukast), antihistamine (Loratadine), cardiac glycoside (Digoxin) and antimalarial (Mefloquine). Mefloquine (an antimalarial drug) may compete with spike protein for binding to ACE2, rather than Hydroxychloroquine, which binds to other region of ACE2 (Table 2 and supplementary Table 2). These above mentioned drugs might find applications to tackle secondary symptoms or complications in COVID-19. Azilsartan kamedoxomil is a potassium salt of azilsartan medoxomil. A recent study predicted binding of azilsartan and zafirlukast to the SARS-CoV-2 binding site [55]. Icatibant, a drug used to treat hereditary angioedema, was recently reported to bind against SARS-CoV-2 binding site [55] as well as the M^pro^ [58].

Several established antiviral and other drugs have been in clinical trials to treat COVID-19. These include remdesivir, lopinavir, ritonavir, ribavirin, oseltamivir, hydroxychloroquine, dexamethasone, etc [1,3,34]. Among these remdesivir seems to bind with high affinities to both ACE1 and ACE2 at sites that do not coincide with SARS-CoV-2 binding (Supplementary Table 2). Clinical trials with remdesivir, an adenosine analog targeted to inhibit RNA dependent RNA polymerase (RdRp) and a much pronounced remedy of COVID-19, has not shown marked clinical improvement in COVID-19 patients [37,60,61]. Binding affinities of 40 different antiviral drugs along with their targets and intended applications are given in supplementary Table 3.

Since the global outbreak of COVID-19, there has been a plethora of reports on drug repurposing for the treatment of COVID-19. These studies have used virtual screening by molecular docking, molecular dynamics simulations or network based approaches to find potential remedies that target different proteins of SARS-CoV-2 [[47], [48], [50], [53], [55], [56], [57], [62], [63]]. Multiple proteins have been described as candidate drug targets, such as the human ACE2 receptor, viral RNA dependent RNA polymerase (RdRp), main protease (M^pro^, also called 3CL^pro^) and papain-like protease (PL^pro^). None of the published reports explored ACE1 as a possible SARS-CoV-2 interacting protein and/or the dugs that might prevent its interaction with the viral protein.

No specific therapeutics for COVID-19 is yet available. A better understanding of the underlying pathobiology will be useful for finding a cure [64]. Till then, already available potential options might be explored to bring comfort to the world. These drugs may be subjected to further analysis to assess their usefulness for the treatment of COVID-19.

## Author contributions

AAS: conceptualization of project; AAS, TA: data curation and analysis, AAS: writing the original draft; AAS, TA: review and editing.

## Statement of ethics

This study neither involved any human or animal, and hence no ethical approval was required.

## Declaration of competing interest

There is no known conflict of interest.

## References

[1] Xie P., Ma W., Tang H., Liu D. (2020). Severe COVID-19: a review of recent progress with a look toward the future. Front Public Health.

[2] Pal M., Berhanu G., Desalegn C., Kandi V. (2020). Severe acute respiratory syndrome coronavirus-2 (SARS-CoV-2): an update. Cureus.

[3] Harapan H., Itoh N., Yufika A., Keam S., Te H., Megawati D. (2020). Coronavirus disease 2019 (COVID-19): a literature review. J Infect Public Health.

[4] Wu Y., Guo C., Tang L., Hong Z., Zhou J., Dong X. (2020). Prolonged presence of SARS-CoV-2 viral RNA in faecal samples. Lancet Gastroenterol Hepatol.

[5] Wrapp D., Wang N., Corbett K.S., Goldsmith J.A., Hsieh C.L., Abiona O. (2020). Cryo-EM structure of the 2019-nCoV spike in the prefusion conformation. Science.

[6] Yan R., Zhang Y., Li Y., Xia L., Guo Y., Zhou Q. (2020). Structural basis for the recognition of SARS-CoV-2 by full-length human ACE2. Science.

[7] Wan Y., Shang J., Graham R., Baric R.S., Li F. (2020). Receptor recognition by the novel Coronavirus from Wuhan: an analysis based on decade-long structural studies of SARS Coronavirus. J. Virol..

[8] Nguyen H.L., Lan P.D., Thai N.Q., Nissley D.A., Ep O.B., Li M.S. (2020). Does SARS-CoV-2 bind to human ACE2 more strongly than does SARS-CoV?. J. Phys. Chem. B.

[9] Xu P., Zhou Q., Xu J. (2020). Mechanism of thrombocytopenia in COVID-19 patients. Ann. Hematol..

[10] Xu X., Chen P., Wang J., Feng J., Zhou H., Li X. (2020). Evolution of the novel coronavirus from the ongoing Wuhan outbreak and modeling of its spike protein for risk of human transmission. Sci. China Life Sci..

[11] Lubbe L., Cozier G.E., Oosthuizen D., Acharya K.R., Sturrock E.D. (2020). ACE2 and ACE: structure-based insights into mechanism, regulation and receptor recognition by SARS-CoV. Clin. Sci. (Lond.).

[12] Guang C., Phillips R.D., Jiang B., Milani F. (2012). Three key proteases--angiotensin-I-converting enzyme (ACE), ACE2 and renin--within and beyond the renin-angiotensin system. Arch Cardiovasc Dis.

[13] Guy J.L., Jackson R.M., Acharya K.R., Sturrock E.D., Hooper N.M., Turner A.J. (2003). Angiotensin-converting enzyme-2 (ACE2): comparative modeling of the active site, specificity requirements, and chloride dependence. Biochemistry.

[14] Raizada M.K., Ferreira A.J. (2007). ACE2: a new target for cardiovascular disease therapeutics. J. Cardiovasc. Pharmacol..

[15] Clarke N.E., Turner A.J. (2012). Angiotensin-converting enzyme 2: the first decade. Int. J. Hypertens..

[16] Aguiar J.A., Tremblay B.J., Mansfield M.J., Woody O., Lobb B., Banerjee A. (2020). Gene expression and in situ protein profiling of candidate SARS-CoV-2 receptors in human airway epithelial cells and lung tissue. Eur. Respir. J..

[17] Hatami N., Ahi S., Sadeghinikoo A., Foroughian M., Javdani F., Kalani N. (2020). Worldwide ACE (I/D) polymorphism may affect COVID-19 recovery rate: an ecological meta-regression. Endocrine.

[18] Yamamoto N., Ariumi Y., Nishida N., Yamamoto R., Bauer G., Gojobori T. (2020). SARS-CoV-2 infections and COVID-19 mortalities strongly correlate with ACE1 I/D genotype. Gene.

[19] Kruse R.L. (2020). Therapeutic strategies in an outbreak scenario to treat the novel coronavirus originating in Wuhan, China.

[20] Natesh R., Schwager S.L., Sturrock E.D., Acharya K.R. (2003). Crystal structure of the human angiotensin-converting enzyme-lisinopril complex. Nature.

[21] Wang Q., Zhang Y., Wu L., Niu S., Song C., Zhang Z. (2020). Structural and functional basis of SARS-CoV-2 entry by using human ACE2. Cell.

[22] Walls A.C., Park Y.J., Tortorici M.A., Wall A., McGuire A.T., Veesler D. (2020). Structure, function, and antigenicity of the SARS-CoV-2 spike glycoprotein. Cell.

[23] Berman H.M., Westbrook J., Feng Z., Gilliland G., Bhat T.N., Weissig H. (2000). The protein Data Bank. Nucleic Acids Res..

[24] Dassault Systèmes Biovia Corp (2020). Discovery Studio Visualizer v20.1.0.19295. San Diego, USA.

[25] Källberg M., Margaryan G., Wang S., Ma J., Xu J. (2014). RaptorX server: a resource for template-based protein structure modeling. Methods Mol. Biol..

[26] McNicholas S., Potterton E., Wilson K.S., Noble M.E. (2011). Presenting your structures: the CCP4mg molecular-graphics software. Acta Crystallogr D Biol Crystallogr.

[27] van Zundert G.C.P., Rodrigues J., Trellet M., Schmitz C., Kastritis P.L., Karaca E. (2016). The HADDOCK2.2 web server: user-friendly integrative modeling of biomolecular complexes. J. Mol. Biol..

[28] Delano W.L. (2004). Use of PyMOL as a communications tool for molecular science. Am Chem Soc.

[29] Wishart D.S., Feunang Y.D., Guo A.C., Lo E.J., Marcu A., Grant J.R. (2018). DrugBank 5.0: a major update to the DrugBank database for 2018. Nucleic Acids Res..

[30] Gilson M.K., Liu T., Baitaluk M., Nicola G., Hwang L., Chong J. (2016). BindingDB in 2015: a public database for medicinal chemistry, computational chemistry and systems pharmacology. Nucleic Acids Res..

[31] Douguet D. (2018). Data sets representative of the structures and experimental properties of FDA-approved drugs. ACS Med. Chem. Lett..

[32] Trott O., Olson A.J. (2010). AutoDock Vina: improving the speed and accuracy of docking with a new scoring function, efficient optimization, and multithreading. J. Comput. Chem..

[33] Dallakyan S., Olson A.J. (2015). Small-molecule library screening by docking with PyRx. Methods Mol. Biol..

[34] Jiang F., Deng L., Zhang L., Cai Y., Cheung C.W., Xia Z. (2020). Review of the clinical characteristics of coronavirus disease 2019 (COVID-19). J. Gen. Intern. Med..

[35] Hamming I., Timens W., Bulthuis M.L.C., Lely A.T., Navis G.J., van Goor H. (2004). Tissue distribution of ACE2 protein, the functional receptor for SARS coronavirus. A first step in understanding SARS pathogenesis. J. Pathol..

[36] Sayers E.W., Beck J., Brister J.R., Bolton E.E., Canese K., Comeau D.C. (2020). Database resources of the national center for biotechnology information. Nucleic Acids Res..

[37] Altay O., Mohammadi E., Lam S., Turkez H., Boren J., Nielsen J. (2020). Current status of COVID-19 therapies and drug repositioning applications. iScience.

[38] March-Vila E., Pinzi L., Sturm N., Tinivella A., Engkvist O., Chen H. (2017). On the integration of in silico drug design methods for drug repurposing. Front. Pharmacol..

[39] Pinzi L., Rastelli G. (2019). Molecular docking: shifting paradigms in drug discovery. Int. J. Mol. Sci..

[40] Kinnings S.L., Liu N., Buchmeier N., Tonge P.J., Xie L., Bourne P.E. (2009). Drug discovery using chemical systems biology: repositioning the safe medicine Comtan to treat multi-drug and extensively drug resistant tuberculosis. PLoS Comput. Biol..

[41] Li Y.Y., An J., Jones S.J.M. (2011). A computational approach to finding novel targets for existing drugs. PLoS Comput. Biol..

[42] Dakshanamurthy S., Issa N.T., Assefnia S., Seshasayee A., Peters O.J., Madhavan S. (2012). Predicting new indications for approved drugs usinga proteo-chemometric method. J. Med. Chem..

[43] Pagadala N.S., Syed K., Tuszynski J. (2017). Software for molecular docking: a review. Biophys Rev.

[44] Kim S., Chen J., Cheng T., Gindulyte A., He J., He S. (2019). PubChem 2019 update: improved access to chemical data. Nucleic Acids Res..

[45] Ahmad I., Rathore F.A. (2020). Neurological manifestations and complications of COVID-19: a literature review. J. Clin. Neurosci..

[46] Li X., Geng M., Peng Y., Meng L., Lu S. (2020). Molecular immune pathogenesis and diagnosis of COVID-19. J Pharm Anal.

[47] Li Z., Li X., Huang Y.-Y., Wu Y., Liu R., Zhou L. (2020). Identify potent SARS-CoV-2 main protease inhibitors via accelerated free energy perturbation-based virtual screening of existing drugs. Proc. Natl. Acad. Sci. Unit. States Am..

[48] Loucera C., Esteban-Medina M., Rian K., Falco M.M., Dopazo J., Peña-Chilet M. (2020). Drug repurposing for COVID-19 using machine learning and mechanistic models of signal transduction circuits related to SARS-CoV-2 infection. Signal Transduct Target Ther.

[49] Zhou P., Liu Z., Chen Y., Xiao Y., Huang X., Fan X.-G. (2020). Bacterial and fungal infections in COVID-19 patients: a matter of concern. Infect. Contr. Hosp. Epidemiol..

[50] Zhou Y., Hou Y., Shen J., Huang Y., Martin W., Cheng F. (2020). Network-based drug repurposing for novel coronavirus 2019-nCoV/SARS-CoV-2. Cell Discov.

[51] Giannis D., Ziogas I.A., Gianni P. (2020). Coagulation disorders in coronavirus infected patients: COVID-19, SARS-CoV-1, MERS-CoV and lessons from the past. J. Clin. Virol..

[52] Biswas S., Thakur V., Kaur P., Khan A., Kulshrestha S., Kumar P. (2021). Blood clots in COVID-19 patients: simplifying the curious mystery. Med. Hypotheses.

[53] Choudhary S., Malik Y.S., Tomar S. (2020). Identification of SARS-CoV-2 cell entry inhibitors by drug repurposing using in silico structure-based virtual screening approach. Front. Immunol..

[54] Patel A.B., Verma A. (2020). COVID-19 and angiotensin-converting enzyme inhibitors and angiotensin receptor blockers: what is the evidence?. J. Am. Med. Assoc..

[55] Maffucci I., Contini A. (2020). In silico drug repurposing for SARS-CoV-2 main proteinase and spike proteins. J. Proteome Res..

[56] Indu P., Rameshkumar M.R., Arunagirinathan N., Al-Dhabi N.A., Valan Arasu M., Ignacimuthu S. (2020). Raltegravir, Indinavir, Tipranavir, Dolutegravir, and Etravirine against main protease and RNA-dependent RNA polymerase of SARS-CoV-2: a molecular docking and drug repurposing approach. J Infect Public Health.

[57] Koulgi S., Jani V., Uppuladinne M., Sonavane U., Nath A.K., Darbari H. (2020). Drug repurposing studies targeting SARS-CoV-2: an ensemble docking approach on drug target 3C-like protease (3CL^pro^). J. Biomol. Struct. Dyn..

[58] Mohamed K., Yazdanpanah N., Saghazadeh A., Rezaei N. (2021). Computational drug discovery and repurposing for the treatment of COVID-19: a systematic review. Bioorg. Chem..

[59] Rawson T.M., Moore L.S.P., Zhu N., Ranganathan N., Skolimowska K., Gilchrist M. (2020). Bacterial and fungal co-infection in individuals with coronavirus: a rapid review to support COVID-19 antimicrobial prescribing. Clin. Infect. Dis..

[60] Martinez M.A. (2020). Clinical trials of repurposed antivirals for SARS-CoV-2. Antimicrob. Agents Chemother..

[61] Yoo J.H. (2020). Uncertainty about the efficacy of remdesivir on COVID-19. J. Kor. Med. Sci..

[62] Deshpande R.R., Tiwari A.P., Nyayanit N., Modak M. (2020). In silico molecular docking analysis for repurposing therapeutics against multiple proteins from SARS-CoV-2. Eur. J. Pharmacol..

[63] Glebov O.O. (2020). Understanding SARS-CoV-2 endocytosis for COVID-19 drug repurposing. FEBS J..

[64] Zhang H., Penninger J.M., Li Y., Zhong N., Slutsky A.S. (2020). Angiotensin-converting enzyme 2 (ACE2) as a SARS-CoV-2 receptor: molecular mechanisms and potential therapeutic target. Intensive Care Med..

