# Supplementary material for: Repurposing of approved drugs with potential to interact with SARS-CoV-2 receptor
Source: Biochem Biophys Rep. 2021 Mar 29;26:100982. doi: 10.1016/j.bbrep.2021.100982 (PMC8006196; doi:10.1016/j.bbrep.2021.100982)
Supplement: Multimedia component 1 [file mmc1.doc]

**Supplementary table 1:** List of interacting amino acids of SARS-CoV-2 spike protein with ACE1 and ACE2.

| **Receptor-Ligand** | **Category** | **Types** | **From*** | **From chemistry** | **To*** | **To chemistry** | **Distance** |
| --- | --- | --- | --- | --- | --- | --- | --- |
| ACE1 and  S protein SARS-CoV-2 | Hydrogen Bond; Electrostatic | Salt Bridge; Attractive Charge | A:LYS84 | H-Donor; Positive | B:ASP405 | H-Acceptor; Negative | 1.5761 |
|  | Hydrogen Bond; Electrostatic | Salt Bridge; Attractive Charge | B:ARG408 | H-Donor; Positive | A:GLU61 | H-Acceptor; Negative | 1.94494 |
|  | Hydrogen Bond | Conventional Hydrogen Bond | A:GLN87 | H-Donor | B:ASP405 | H-Acceptor | 1.78862 |
|  | Hydrogen Bond | Conventional Hydrogen Bond | A:LYS94 | H-Donor | B:ASN501 | H-Acceptor | 1.64556 |
|  | Hydrogen Bond | Conventional Hydrogen Bond | A:LYS363 | H-Donor | B:ARG457 | H-Acceptor | 2.49257 |
|  | Hydrogen Bond | Conventional Hydrogen Bond | A:LYS363 | H-Donor | B:ARG457 | H-Acceptor | 2.31517 |
|  | Hydrogen Bond | Conventional Hydrogen Bond | B:ARG403 | H-Donor | A:GLN87 | H-Acceptor | 2.47327 |
|  | Hydrogen Bond | Conventional Hydrogen Bond | B:GLN409 | H-Donor | A:GLU61 | H-Acceptor | 1.93265 |
|  | Hydrogen Bond | Conventional Hydrogen Bond | B:LYS417 | H-Donor | A:GLU64 | H-Acceptor | 2.6966 |
|  | Hydrogen Bond | Conventional Hydrogen Bond | B:LYS417 | H-Donor | A:GLN56 | H-Acceptor | 1.73257 |
|  | Hydrogen Bond | Conventional Hydrogen Bond | B:TYR421 | H-Donor | A:GLU334 | H-Acceptor | 2.00324 |
|  | Hydrogen Bond | Conventional Hydrogen Bond | B:TYR449 | H-Donor | A:GLU50 | H-Acceptor | 1.68 |
|  | Hydrogen Bond | Conventional Hydrogen Bond | B:TYR453 | H-Donor | A:ARG53 | H-Acceptor | 2.37109 |
|  | Hydrogen Bond | Conventional Hydrogen Bond | B:GLN493 | H-Donor | A:ASP52 | H-Acceptor | 2.34209 |
|  | Hydrogen Bond | Conventional Hydrogen Bond | B:TYR505 | H-Donor | A:THR54 | H-Acceptor | 2.14325 |
|  | Hydrogen Bond | Carbon Hydrogen Bond | B:ARG408 | H-Donor | A:GLU61 | H-Acceptor | 3.49064 |
|  | Hydrogen Bond | Carbon Hydrogen Bond | B:GLY416 | H-Donor | A:GLU64 | H-Acceptor | 3.09962 |
|  | Hydrogen Bond | Carbon Hydrogen Bond | B:LYS417 | H-Donor | A:ASN60 | H-Acceptor | 3.26541 |
|  | Hydrophobic | Pi-Pi Stacked | A:HIS91 | Pi-Orbitals | B:TYR505 | Pi-Orbitals | 5.51939 |
|  | Hydrophobic | Pi-Alkyl | B:TYR453 | Pi-Orbitals | A:VAL57 | Alkyl | 5.47248 |
|  | Hydrophobic | Pi-Alkyl | B:TYR505 | Pi-Orbitals | A:VAL58 | Alkyl | 5.0717 |
|  | Hydrogen Bond; Electrostatic | Salt Bridge; Attractive Charge | A:LYS84 | H-Donor; Positive | B:ASP405 | H-Acceptor; Negative | 1.5761 |
|  | Hydrogen Bond; Electrostatic | Salt Bridge; Attractive Charge | B:ARG408 | H-Donor; Positive | A:GLU61 | H-Acceptor; Negative | 1.94494 |
|  | Hydrogen Bond | Conventional Hydrogen Bond | A:GLN87 | H-Donor | B:ASP405 | H-Acceptor | 1.78862 |
|  | Hydrogen Bond | Conventional Hydrogen Bond | A:LYS94 | H-Donor | B:ASN501 | H-Acceptor | 1.64556 |
|  | Hydrogen Bond | Conventional Hydrogen Bond | A:LYS363 | H-Donor | B:ARG457 | H-Acceptor | 2.49257 |
|  | Hydrogen Bond | Conventional Hydrogen Bond | A:LYS363 | H-Donor | B:ARG457 | H-Acceptor | 2.31517 |
|  | Hydrogen Bond | Conventional Hydrogen Bond | B:ARG403 | H-Donor | A:GLN87 | H-Acceptor | 2.47327 |
|  | Hydrogen Bond | Conventional Hydrogen Bond | B:GLN409 | H-Donor | A:GLU61 | H-Acceptor | 1.93265 |
|  | Hydrogen Bond | Conventional Hydrogen Bond | B:LYS417 | H-Donor | A:GLU64 | H-Acceptor | 2.6966 |
|  | Hydrogen Bond | Conventional Hydrogen Bond | B:LYS417 | H-Donor | A:GLN56 | H-Acceptor | 1.73257 |
|  | Hydrogen Bond | Conventional Hydrogen Bond | B:TYR421 | H-Donor | A:GLU334 | H-Acceptor | 2.00324 |
|  | Hydrogen Bond | Conventional Hydrogen Bond | B:TYR449 | H-Donor | A:GLU50 | H-Acceptor | 1.68 |
|  | Hydrogen Bond | Conventional Hydrogen Bond | B:TYR453 | H-Donor | A:ARG53 | H-Acceptor | 2.37109 |
|  | Hydrogen Bond | Conventional Hydrogen Bond | B:GLN493 | H-Donor | A:ASP52 | H-Acceptor | 2.34209 |
|  | Hydrogen Bond | Conventional Hydrogen Bond | B:TYR505 | H-Donor | A:THR54 | H-Acceptor | 2.14325 |
|  | Hydrogen Bond | Carbon Hydrogen Bond | B:ARG408 | H-Donor | A:GLU61 | H-Acceptor | 3.49064 |
|  | Hydrogen Bond | Carbon Hydrogen Bond | B:GLY416 | H-Donor | A:GLU64 | H-Acceptor | 3.09962 |
|  | Hydrogen Bond | Carbon Hydrogen Bond | B:LYS417 | H-Donor | A:ASN60 | H-Acceptor | 3.26541 |
|  | Hydrophobic | Pi-Pi Stacked | A:HIS91 | Pi-Orbitals | B:TYR505 | Pi-Orbitals | 5.51939 |
|  | Hydrophobic | Pi-Alkyl | B:TYR453 | Pi-Orbitals | A:VAL57 | Alkyl | 5.47248 |
|  | Hydrophobic | Pi-Alkyl | B:TYR505 | Pi-Orbitals | A:VAL58 | Alkyl | 5.0717 |
|  | Hydrogen Bond; Electrostatic | Salt Bridge; Attractive Charge | A:LYS84 | H-Donor; Positive | B:ASP405 | H-Acceptor; Negative | 1.5761 |
|  | Hydrogen Bond; Electrostatic | Salt Bridge; Attractive Charge | B:ARG408 | H-Donor; Positive | A:GLU61 | H-Acceptor; Negative | 1.94494 |
|  | Hydrogen Bond | Conventional Hydrogen Bond | A:GLN87 | H-Donor | B:ASP405 | H-Acceptor | 1.78862 |
|  | Hydrogen Bond | Conventional Hydrogen Bond | A:LYS94 | H-Donor | B:ASN501 | H-Acceptor | 1.64556 |
|  | Hydrogen Bond | Conventional Hydrogen Bond | A:LYS363 | H-Donor | B:ARG457 | H-Acceptor | 2.49257 |
|  | Hydrogen Bond | Conventional Hydrogen Bond | A:LYS363 | H-Donor | B:ARG457 | H-Acceptor | 2.31517 |
|  | Hydrogen Bond | Conventional Hydrogen Bond | B:ARG403 | H-Donor | A:GLN87 | H-Acceptor | 2.47327 |
|  | Hydrogen Bond | Conventional Hydrogen Bond | B:GLN409 | H-Donor | A:GLU61 | H-Acceptor | 1.93265 |
|  | Hydrogen Bond | Conventional Hydrogen Bond | B:LYS417 | H-Donor | A:GLU64 | H-Acceptor | 2.6966 |
|  | Hydrogen Bond | Conventional Hydrogen Bond | B:LYS417 | H-Donor | A:GLN56 | H-Acceptor | 1.73257 |
|  | Hydrogen Bond | Conventional Hydrogen Bond | B:TYR421 | H-Donor | A:GLU334 | H-Acceptor | 2.00324 |
|  | Hydrogen Bond | Conventional Hydrogen Bond | B:TYR449 | H-Donor | A:GLU50 | H-Acceptor | 1.68 |
|  | Hydrogen Bond | Conventional Hydrogen Bond | B:TYR453 | H-Donor | A:ARG53 | H-Acceptor | 2.37109 |
|  | Hydrogen Bond | Conventional Hydrogen Bond | B:GLN493 | H-Donor | A:ASP52 | H-Acceptor | 2.34209 |
|  | Hydrogen Bond | Conventional Hydrogen Bond | B:TYR505 | H-Donor | A:THR54 | H-Acceptor | 2.14325 |
|  | Hydrogen Bond | Carbon Hydrogen Bond | B:ARG408 | H-Donor | A:GLU61 | H-Acceptor | 3.49064 |
|  | Hydrogen Bond | Carbon Hydrogen Bond | B:GLY416 | H-Donor | A:GLU64 | H-Acceptor | 3.09962 |
|  | Hydrogen Bond | Carbon Hydrogen Bond | B:LYS417 | H-Donor | A:ASN60 | H-Acceptor | 3.26541 |
|  | Hydrophobic | Pi-Pi Stacked | A:HIS91 | Pi-Orbitals | B:TYR505 | Pi-Orbitals | 5.51939 |
|  | Hydrophobic | Pi-Alkyl | B:TYR453 | Pi-Orbitals | A:VAL57 | Alkyl | 5.47248 |
|  | Hydrophobic | Pi-Alkyl | B:TYR505 | Pi-Orbitals | A:VAL58 | Alkyl | 5.0717 |
| ACE2 and  S protein SARS-CoV-2 | Hydrogen Bond; Electrostatic | Salt Bridge; Attractive Charge | B:LYS417 | H-Donor; Positive | A:ASP30 | H-Acceptor; Negative | 2.53243 |
|  | Hydrogen Bond | Conventional Hydrogen Bond | A:LYS68 | H-Donor | B:GLN498 | H-Acceptor | 1.76044 |
|  | Hydrogen Bond | Conventional Hydrogen Bond | A:TYR83 | H-Donor | B:PHE490 | H-Acceptor | 1.79837 |
|  | Hydrogen Bond | Conventional Hydrogen Bond | B:TYR449 | H-Donor | A:LYS68 | H-Acceptor | 1.87151 |
|  | Hydrogen Bond | Conventional Hydrogen Bond | B:GLY496 | H-Donor | A:GLU35 | H-Acceptor | 2.3377 |
|  | Hydrogen Bond | Conventional Hydrogen Bond | B:ASN501 | H-Donor | A:GLU35 | H-Acceptor | 2.70323 |
|  | Other | Pi-Sulfur | A:MET82 | Sulfur | B:PHE490 | Pi-Orbitals | 4.87547 |
|  | Hydrogen Bond; Electrostatic | Salt Bridge; Attractive Charge | B:LYS417 | H-Donor; Positive | A:ASP30 | H-Acceptor; Negative | 2.53243 |
|  | Hydrogen Bond | Conventional Hydrogen Bond | A:LYS68 | H-Donor | B:GLN498 | H-Acceptor | 1.76044 |
|  | Hydrogen Bond | Conventional Hydrogen Bond | A:TYR83 | H-Donor | B:PHE490 | H-Acceptor | 1.79837 |
|  | Hydrogen Bond | Conventional Hydrogen Bond | B:TYR449 | H-Donor | A:LYS68 | H-Acceptor | 1.87151 |
|  | Hydrogen Bond | Conventional Hydrogen Bond | B:GLY496 | H-Donor | A:GLU35 | H-Acceptor | 2.3377 |
|  | Hydrogen Bond | Conventional Hydrogen Bond | B:ASN501 | H-Donor | A:GLU35 | H-Acceptor | 2.70323 |
|  | Other | Pi-Sulfur | A:MET82 | Sulfur | B:PHE490 | Pi-Orbitals | 4.87547 |
| *A= receptor (ACE1/ACE2), B= ligand (S protein) | | | | | | | |
