# Supplementary material for: Repurposing of approved drugs with potential to interact with SARS-CoV-2 receptor
Source: Biochem Biophys Rep. 2021 Mar 29;26:100982. doi: 10.1016/j.bbrep.2021.100982 (PMC8006196; doi:10.1016/j.bbrep.2021.100982)
Supplement: Multimedia component 2 [file mmc2.docx]

Supplementary table 2: List of drugs and their binding energies to ACE1 and ACE2.

| **Ligand** | **Human ACE1** | **Human ACE2** |
| --- | --- | --- |
|  | **Binding energy (kcal/mol)** | **Binding energy (kcal/mol)** |
| (S)-NICARDIPINE | -8.6 | -6.0 |
| (S)-NITRENDIPINE | -7.5 | -6.5 |
| 17-BETA ESTRADIOL | -8.2 | -5.7 |
| 4-AMINOPYRIDINE | -3.9 | -4.0 |
| 4-PHENYLAMINO-3-QUINOLINECARBONITRILE | -8.2 | -6.5 |
| ABACAVIR | -8.2 | -6.3 |
| ABARELIX | -6.0 | -4.4 |
| ABEMACICLIB | -9.1 | -6.7 |
| ABIRATERONE ACETATE | -9.8 | -7.0 |
| ACALABRUTINIB | -9.6 | -7.2 |
| ACAMPROSATE | -5.7 | -4.5 |
| ACARBOSE | -8.2 | -6.0 |
| ACEBUTOLOL | -7.3 | -5.6 |
| ACETAMINOPHEN | -5.5 | -5.6 |
| ACETAZOLAMIDE | -6.2 | -5.1 |
| ACETOHEXAMIDE | -8.6 | -6.3 |
| ACETOHYDROXAMIC ACID | -3.9 | -3.5 |
| ACETOPHENAZINE | -8.0 | -6.2 |
| ACETYLCHOLINE | -4.3 | -3.5 |
| ACETYLCYSTEINE | -4.7 | -3.9 |
| ACETYLDIGITOXIN | -10.0 | -7.1 |
| ACRISORCIN | -5.9 | -5.8 |
| ACYCLOVIR | -6.7 | -5.2 |
| ADAPALENE | -9.2 | -7.3 |
| ADEFOVIR DIPIVOXIL | -7.6 | -4.8 |
| ADENOSINE | -7.0 | -5.5 |
| ALATROFLOXACIN | -10.6 | -6.4 |
| ALBENDAZOLE | -6.9 | -5.2 |
| ALBUTEROL | -6.6 | -5.2 |
| ALCAFTADINE | -9.0 | -5.8 |
| ALCLOMETASONE DIPROPIONATE | -8.6 | -6.5 |
| ALFENTANIL | -7.4 | -4.9 |
| ALFUZOSIN | -7.7 | -5.6 |
| ALISKIREN | -7.5 | -5.9 |
| ALITRETINOIN | -7.4 | -5.6 |
| ALLOPURINOL | -5.6 | -5.1 |
| ALMOTRIPTAN | -7.0 | -6.0 |
| ALOSETRON | -8.3 | -6.4 |
| ALPELISIB | -8.7 | -6.6 |
| ALPHA-TOCOPHEROL | -7.4 | -5.8 |
| ALPHA-TOCOPHEROL ACETATE | -7.8 | -5.6 |
| ALPRAZOLAM | -8.6 | -5.9 |
| ALPROSTADIL | -6.2 | -5.3 |
| ALTRETAMINE | -5.1 | -4.1 |
| ALVIMOPAN | -8.3 | -6.4 |
| AMANTADINE | -5.5 | -4.6 |
| AMBENONIUM | -7.6 | -5.5 |
| AMBRISENTAN | -7.8 | -5.2 |
| AMCINONIDE | -10.7 | -7.4 |
| AMDINOCILLIN | -7.1 | -5.8 |
| AMIFAMPRIDINE | -4.1 | -4.2 |
| AMIFOSTINE | -4.8 | -3.8 |
| AMIKACIN | -7.2 | -5.4 |
| AMILORIDE | -6.4 | -5.9 |
| AMINOCAPROIC ACID | -4.9 | -4.4 |
| AMINOGLUTETHIMIDE | -7.4 | -5.7 |
| AMINOLEVULINIC ACID | -4.7 | -5.3 |
| AMINOMETHYLBENZOIC ACID | -6.4 | -3.9 |
| AMINOPTERIN | -9.0 | -5.1 |
| AMINOSALICYLIC ACID | -6.1 | -5.4 |
| AMIODARONE | -7.4 | -5.8 |
| AMITRIPTYLINE | -7.7 | -6.7 |
| AMLEXANOX | -8.7 | -4.4 |
| AMLODIPINE | -6.7 | -4.2 |
| AMODIAQUINE | -7.5 | -6.9 |
| AMOXAPINE | -8.8 | -5.5 |
| AMOXICILLIN | -8.2 | -6.2 |
| AMPHETAMINE | -5.3 | -5.2 |
| AMPHOTERICIN B | -7.1 | -6.0 |
| AMPICILLIN | -8.1 | -5.8 |
| AMPRENAVIR | -7.5 | -5.1 |
| ANAGRELIDE | -7.2 | -5.7 |
| ANASTROZOLE | -8.0 | -6.0 |
| ANGIOTENSIN II | -9.5 | -6.1 |
| ANIDULAFUNGIN | -6.6 | -5.7 |
| ANILERIDINE | -8.3 | -6.0 |
| ANISINDIONE | -7.5 | -5.5 |
| ANTAZOLINE | -7.4 | -5.1 |
| APALUTAMIDE | -10.1 | -6.8 |
| APOMORPHINE | -8.1 | -5.8 |
| APRACLONIDINE | -6.5 | -5.5 |
| APREPITANT | -8.3 | -6.3 |
| ARBUTAMINE | -7.6 | -5.9 |
| ARFORMOTEROL | -7.4 | -6.3 |
| ARGATROBAN | -9.4 | -6.0 |
| ARIPIPRAZOLE | -8.8 | -6.5 |
| ARTEMETHER | -8.4 | -5.4 |
| ASCORBIC ACID | -5.9 | -4.8 |
| ASENAPINE | -8.3 | -6.0 |
| ASPIRIN | -6.1 | -4.6 |
| ATAZANAVIR | -8.5 | -6.4 |
| ATENOLOL | -6.0 | -5.3 |
| ATOVAQUONE | -8.9 | -6.8 |
| ATROPINE | -6.9 | -5.2 |
| AVATROMBOPAG | -6.9 | -7.4 |
| AXITINIB | -9.3 | -7.0 |
| AZACITIDINE | -6.6 | -5.1 |
| AZATADINE | -8.0 | -6.0 |
| AZATHIOPRINE | -6.5 | -5.1 |
| AZELAIC ACID | -5.2 | -4.1 |
| AZELASTINE | -9.0 | -6.6 |
| AZILSARTAN KAMEDOXOMIL | -9.6 | -6.4 |
| AZITHROMYCIN | -6.6 | -5.5 |
| AZLOCILLIN | -9.4 | -6.7 |
| AZTREONAM | -7.7 | -6.2 |
| BACAMPICILLIN | -7.7 | -5.6 |
| BACITRACIN | -5.3 | -4.0 |
| BACLOFEN | -6.0 | -6.3 |
| BALOXAVIR | -9.0 | -6.4 |
| BALOXAVIR MARBOXIL | -8.6 | -6.4 |
| BALSALAZIDE | -8.2 | -6.6 |
| BARICITINIB | -8.4 | -5.6 |
| BECLOMETHASONE DIPROPIONATE | -7.8 | -6.6 |
| BENAZEPRIL | -7.5 | -5.9 |
| BENDROFLUMETHIAZIDE | -8.4 | -6.1 |
| BENZHYDROCODONE | -9.0 | -7.0 |
| BENZNIDAZOLE | -7.9 | -6.3 |
| BENZONATATE | -5.9 | -4.8 |
| BENZOYL PEROXIDE | -6.9 | -6.5 |
| BENZPHETAMINE | -6.1 | -5.7 |
| BENZQUINAMIDE | -7.5 | -5.7 |
| BENZTHIAZIDE | -8.4 | -6.3 |
| BENZTROPINE | -7.8 | -5.3 |
| BENZYL ALCOHOL | -5.0 | -4.8 |
| BENZYL BENZOATE | -6.3 | -6.2 |
| BEPOTASTINE | -8.4 | -6.0 |
| BEPRIDIL | -7.1 | -4.7 |
| BESIFLOXACIN | -8.6 | -5.6 |
| BETAINE | -4.0 | -3.2 |
| BETAMETHASONE | -9.3 | -6.4 |
| BETAMETHASONE ACETATE | -9.2 | -6.5 |
| BETAMETHASONE BENZOATE | -10.8 | -6.5 |
| BETAMETHASONE DIPROPIONATE | -8.8 | -5.5 |
| BETAMETHASONE PHOSPHATE | -8.6 | -6.6 |
| BETAMETHASONE VALERATE | -9.7 | -5.7 |
| BETAXOLOL | -6.6 | -5.2 |
| BETAZOLE | -4.4 | -4.6 |
| BETHANECHOL | -4.5 | -3.8 |
| BETRIXABAN | -8.5 | -6.5 |
| BEXAROTENE | -9.2 | -6.4 |
| BICALUTAMIDE | -8.9 | -7.0 |
| BICTEGRAVIR | -9.7 | -7.8 |
| BIMATOPROST | -7.6 | -6.2 |
| BINIMETINIB | -7.6 | -5.5 |
| BIOTIN | -6.4 | -5.5 |
| BIPERIDEN | -8.0 | -6.1 |
| BISOPROLOL | -6.1 | -5.1 |
| BISPHOSPHONATE | -5.8 | -5.5 |
| BITOLTEROL | -8.1 | -5.7 |
| BLEOMYCIN | -4.1 | -4.7 |
| BMS-354825 | -8.6 | -6.5 |
| BOCEPREVIR | -8.1 | -5.4 |
| BOSENTAN | -8.4 | -5.2 |
| BREXANOLONE | -8.5 | -6.0 |
| BRIGATINIB | -8.8 | -6.4 |
| BRIMONIDINE | -7.2 | -5.2 |
| BRINZOLAMIDE | -7.2 | -4.9 |
| BROMFENAC | -7.8 | -5.7 |
| BROMODIPHENHYDRAMINE | -6.8 | -4.7 |
| BROMPHENIRAMINE | -7.0 | -6.2 |
| BRZ | -7.4 | -4.9 |
| BUCLIZINE | -8.9 | -6.1 |
| BUDESONIDE | -9.7 | -6.2 |
| BUMETANIDE | -7.5 | -5.2 |
| BUPIVACAINE | -7.0 | -5.9 |
| BUPRENORPHINE | -7.6 | -5.9 |
| BUPROPION | -6.5 | -5.2 |
| BUSPIRONE | -8.7 | -7.2 |
| BUSULFAN | -5.7 | -4.5 |
| BUTABARBITAL | -6.5 | -4.8 |
| BUTALBITAL | -6.6 | -4.7 |
| BUTENAFINE | -7.4 | -6.1 |
| BUTOCONAZOLE | -6.6 | -4.8 |
| BUTORPHANOL | -7.6 | -6.2 |
| CABAZITAXEL | -5.9 | -5.9 |
| CAFFEINE | -6.0 | -4.9 |
| CALCIFEDIOL | -8.6 | -6.2 |
| CALCIPOTRIENE | -8.7 | -6.7 |
| CANDESARTAN CILEXETIL | -8.9 | -6.7 |
| CANDICIDIN | -6.0 | -6.3 |
| CANNABIDIOL | -7.4 | -5.3 |
| CAPECITABINE | -7.4 | -6.1 |
| CAPREOMYCIN | -8.8 | -6.4 |
| CAPSAICIN | -7.3 | -5.8 |
| CAPTOPRIL | -5.6 | -4.2 |
| CARBACHOL | -4.7 | -3.7 |
| CARBAMAZEPINE | -7.9 | -7.7 |
| CARBENICILLIN | -8.3 | -6.1 |
| CARBENICILLIN INDANYL | -9.4 | -7.1 |
| CARBIDOPA | -6.9 | -5.7 |
| CARBINOXAMINE | -6.7 | -5.9 |
| CARGLUMIC ACID | -5.9 | -5.2 |
| CARISOPRODOL | -6.1 | -5.4 |
| CARMUSTINE | -5.0 | -4.3 |
| CARPHENAZINE | -7.8 | -5.6 |
| CARPROFEN | -8.1 | -6.0 |
| CARTEOLOL | -7.6 | -5.3 |
| CARVEDILOL | -8.4 | -6.1 |
| CEFACLOR | -7.8 | -5.8 |
| CEFADROXIL | -8.0 | -6.4 |
| CEFAMANDOLE NAFATE | -8.3 | -6.3 |
| CEFAZOLIN | -8.2 | -6.4 |
| CEFDINIR | -7.8 | -6.1 |
| CEFDITOREN PIVOXIL | -8.4 | -6.4 |
| CEFIXIME | -7.3 | -6.4 |
| CEFMENOXIME | -7.7 | -6.7 |
| CEFMETAZOLE | -7.4 | -6.0 |
| CEFONICID | -7.4 | -7.1 |
| CEFOPERAZONE | -9.8 | -6.5 |
| CEFORANIDE | -7.8 | -6.9 |
| CEFOTAXIME | -7.4 | -5.9 |
| CEFOTETAN | -8.0 | -6.4 |
| CEFOTIAM | -7.7 | -5.8 |
| CEFOXITIN | -7.1 | -5.5 |
| CEFPIRAMIDE | -9.8 | -6.8 |
| CEFPODOXIME PROXETIL | -8.0 | -5.8 |
| CEFPROZIL | -8.3 | -6.0 |
| CEFTAROLINE | -8.6 | -7.1 |
| CEFTAZIDIME | -7.9 | -6.4 |
| CEFTIBUTEN | -7.4 | -6.2 |
| CEFTIZOXIME | -7.6 | -6.0 |
| CEFTRIAXONE | -8.4 | -7.0 |
| CEFUROXIME | -7.5 | -6.2 |
| CEFUROXIME AXETIL | -8.0 | -5.9 |
| CELECOXIB | -8.3 | -6.5 |
| CEPHALEXIN | -8.0 | -5.9 |
| CEPHALOGLYCIN | -8.1 | -6.0 |
| CEPHALOTHIN | -7.3 | -5.8 |
| CEPHAPIRIN | -7.8 | -6.0 |
| CEPHRADINE | -8.0 | -5.9 |
| CERIVASTATIN | -7.3 | -5.6 |
| CERULETIDE | -6.2 | -6.2 |
| CETIRIZINE | -7.4 | -5.1 |
| CETRORELIX | -9.1 | -6.2 |
| CEVIMELINE | -5.8 | -4.4 |
| CHENODIOL | -8.4 | -6.0 |
| CHLOPHEDIANOL | -7.0 | -4.8 |
| CHLORAMBUCIL | -6.3 | -4.5 |
| CHLORAMPHENICOL | -7.0 | -5.4 |
| CHLORAMPHENICOL PALMITATE | -7.4 | -4.8 |
| CHLORDIAZEPOXIDE | -7.5 | -5.4 |
| CHLORHEXIDINE | -9.5 | -6.9 |
| CHLORMEZANONE | -6.8 | -5.1 |
| CHLOROPROCAINE | -6.5 | -5.1 |
| CHLOROQUINE | -6.7 | -4.9 |
| CHLOROTHIAZIDE | -7.0 | -5.3 |
| CHLOROTRIANISENE | -7.7 | -5.7 |
| CHLOROXINE | -6.4 | -5.4 |
| CHLORPHENESIN CARBAMATE | -6.2 | -5.1 |
| CHLORPHENIRAMINE | -7.0 | -5.7 |
| CHLORPHENTERMINE | -5.5 | -4.8 |
| CHLORPROMAZINE | -7.0 | -5.0 |
| CHLORPROPAMIDE | -6.6 | -6.0 |
| CHLORTETRACYCLINE | -8.3 | -6.5 |
| CHLORTHALIDONE | -8.0 | -6.6 |
| CHLORZOXAZONE | -6.6 | -5.5 |
| CICLESONIDE | -10.4 | -7.0 |
| CICLOPIROX | -6.8 | -6.8 |
| CIDOFOVIR | -6.4 | -5.6 |
| CILASTATIN | -6.4 | -5.5 |
| CILOSTAZOL | -9.0 | -6.5 |
| CIMETIDINE | -6.4 | -4.9 |
| CINACALCET | -7.0 | -5.3 |
| CINOXACIN | -6.8 | -5.4 |
| CIPROFLOXACIN | -8.1 | -5.8 |
| CISATRACURIUM | -7.4 | -4.1 |
| CITALOPRAM | -7.8 | -5.5 |
| CLADRIBINE | -7.2 | -6.4 |
| CLARITHROMYCIN | -8.3 | -4.8 |
| CLAVULANATE | -6.5 | -5.1 |
| CLEMASTINE | -7.4 | -5.0 |
| CLIDINIUM | -7.7 | -6.3 |
| CLINDAMYCIN | -6.8 | -5.7 |
| CLINDAMYCIN PALMITATE | -7.1 | -5.0 |
| CLINDAMYCIN PHOSPHATE | -5.6 | -5.2 |
| CLIOQUINOL | -6.5 | -5.8 |
| CLOBAZAM | -7.9 | -5.7 |
| CLOBETASOL PROPIONATE | -9.8 | -6.5 |
| CLOCORTOLONE PIVALATE | -8.5 | -7.3 |
| CLOFAZIMINE | -9.3 | -6.4 |
| CLOFIBRATE | -6.1 | -4.6 |
| CLOMIPRAMINE | -7.8 | -5.1 |
| CLONAZEPAM | -8.0 | -5.9 |
| CLONIDINE | -6.4 | -5.0 |
| CLOPIDOGREL | -7.5 | -5.4 |
| CLORAZEPATE | -8.1 | -6.1 |
| CLOTRIMAZOLE | -7.4 | -5.2 |
| CLOXACILLIN | -8.4 | -6.7 |
| CLOZAPINE | -8.3 | -6.1 |
| COCAINE | -7.2 | -5.8 |
| CODEINE | -7.7 | -5.9 |
| COLCHICINE | -8.0 | -5.2 |
| COLFOSCERIL PALMITATE | -6.2 | -4.3 |
| COLISTIMETHATE | -5.6 | -3.6 |
| COLISTIN | -5.5 | -4.8 |
| CONIVAPTAN | -10.2 | -7.8 |
| COPANLISIB | -9.0 | -6.8 |
| CORTISONE ACETATE | -8.7 | -6.6 |
| CRIZOTINIB | -9.1 | -7.3 |
| CROMOLYN | -8.7 | -7.1 |
| CROTAMITON | -6.2 | -4.5 |
| CYCLACILLIN | -7.3 | -5.9 |
| CYCLIZINE | -7.7 | -5.5 |
| CYCLOBENZAPRINE | -8.2 | -5.5 |
| CYCLOPENTOLATE | -6.8 | -4.8 |
| CYCLOPHOSPHAMIDE | -5.2 | -4.1 |
| CYCLOSPORINE | -5.1 | -4.5 |
| CYCLOTHIAZIDE | -9.1 | -6.0 |
| CYCRIMINE | -7.6 | -6.8 |
| CYPROHEPTADINE | -9.4 | -6.0 |
| CYSTEAMINE | -2.6 | -2.2 |
| CYTARABINE | -6.4 | -4.9 |
| DABIGATRAN ETEXILATE | -8.9 | -5.7 |
| DACARBAZINE | -5.8 | -5.4 |
| DACOMITINIB | -9.9 | -6.7 |
| DACTINOMYCIN | -7.7 | -4.2 |
| DALFAMPRIDINE | -3.9 | -4.0 |
| DALFOPRISTIN | -9.0 | -6.8 |
| DANAZOL | -9.1 | -7.1 |
| DANTROLENE | -8.4 | -6.4 |
| DAPIPRAZOLE | -8.2 | -6.8 |
| DAPSONE | -7.4 | -5.4 |
| DAPTOMYCIN | -4.3 | -3.5 |
| DARIFENACIN | -9.5 | -6.7 |
| DARUNAVIR | -8.8 | -5.4 |
| DASATINIB | -8.9 | -6.6 |
| DAUNORUBICIN | -9.8 | -6.2 |
| DECITABINE | -6.5 | -5.9 |
| DEFERASIROX | -9.4 | -7.1 |
| DEFERIPRONE | -5.4 | -4.6 |
| DEFEROXAMINE | -7.1 | -6.3 |
| DEFLAZACORT | -9.3 | -6.9 |
| DEGARELIX | -4.8 | -5.2 |
| DELAFLOXACIN | -8.6 | -6.5 |
| DELAVIRDINE | -8.6 | -6.6 |
| DEMECARIUM | -8.0 | -5.3 |
| DEMECLOCYCLINE | -8.3 | -6.3 |
| DESERPIDINE | -9.0 | -6.6 |
| DESFLURANE | -4.9 | -4.7 |
| DESIPRAMINE | -7.4 | -5.5 |
| DESLANOSIDE | -7.5 | -7.9 |
| DESLORATADINE | -8.3 | -6.0 |
| DESOGESTREL | -7.9 | -5.7 |
| DESONIDE | -9.9 | -6.6 |
| DESOXIMETASONE | -9.6 | -6.4 |
| DESOXYCORTICOSTERONE ACETATE | -8.3 | -6.7 |
| DESOXYCORTICOSTERONE PIVALATE | -8.6 | -6.8 |
| DESVENLAFAXINE | -6.9 | -5.2 |
| DEXAMETHASONE | -9.3 | -6.6 |
| DEXBROMPHENIRAMINE | -6.9 | -4.8 |
| DEXCHLORPHENIRAMINE | -6.9 | -6.0 |
| DEXMEDETOMIDINE | -6.5 | -4.9 |
| DEXMETHYLPHENIDATE | -6.8 | -5.3 |
| DEXPANTHENOL | -5.3 | -4.3 |
| DEXRAZOXANE | -7.0 | -6.2 |
| DEXTROTHYROXINE | -6.3 | -5.2 |
| DEZOCINE | -8.8 | -5.6 |
| DIAZEPAM | -7.9 | -5.8 |
| DIAZOXIDE | -6.7 | -5.1 |
| DIBUCAINE | -7.5 | -5.6 |
| DICHLORPHENAMIDE | -7.0 | -4.7 |
| DICLOFENAC | -6.9 | -6.0 |
| DICLOXACILLIN | -8.1 | -6.1 |
| DICUMAROL | -8.5 | -6.4 |
| DICYCLOMINE | -6.5 | -5.1 |
| DIDANOSINE | -7.2 | -5.7 |
| DIENESTROL | -7.3 | -5.5 |
| DIENOGEST | -8.5 | -6.7 |
| DIETHYLCARBAMAZINE | -5.2 | -3.9 |
| DIETHYLPROPION | -6.2 | -4.3 |
| DIETHYLSTILBESTROL | -6.9 | -5.5 |
| DIFENOXIN | -9.0 | -6.6 |
| DIFLORASONE DIACETATE | -8.4 | -6.5 |
| DIFLUNISAL | -7.7 | -6.3 |
| DIFLUPREDNATE | -8.9 | -6.4 |
| DIGITOXIN | -11.2 | -8.0 |
| DIGOXIN | -7.7 | -7.4 |
| DIHYDROCODEINE | -7.3 | -5.8 |
| DIHYDROERGOTAMINE | -12.7 | -7.4 |
| DILTIAZEM | -7.1 | -4.9 |
| DIMENHYDRINATE | -6.2 | -4.7 |
| DIMERCAPROL | -3.1 | -2.7 |
| DINOPROST | -7.0 | -5.0 |
| DINOPROSTONE | -6.7 | -4.5 |
| DIPHEMANIL | -8.0 | -6.1 |
| DIPHENHYDRAMINE | -6.8 | -4.5 |
| DIPHENIDOL | -7.2 | -5.1 |
| DIPHENOXYLATE | -8.4 | -6.5 |
| DIPHENYLPYRALINE | -7.5 | -7.0 |
| DIPIVEFRIN | -7.4 | -5.5 |
| DIPYRIDAMOLE | -7.0 | -4.6 |
| DISOPYRAMIDE | -7.2 | -5.1 |
| DISULFIRAM | -4.6 | -3.5 |
| DOCETAXEL | -9.4 | -6.5 |
| DOCOSANOL | -5.5 | -3.8 |
| DOFETILIDE | -7.6 | -5.9 |
| DOLASETRON | -8.6 | -6.2 |
| DONEPEZIL | -9.1 | -6.9 |
| DOPAMINE | -5.9 | -5.3 |
| DORAVIRINE | -8.5 | -6.5 |
| DORIPENEM | -7.8 | -6.5 |
| DORZOLAMIDE | -7.1 | -5.6 |
| DOXACURIUM | -7.1 | -5.0 |
| DOXAPRAM | -8.0 | -5.3 |
| DOXAZOSIN | -9.6 | -6.7 |
| DOXEPIN | -8.1 | -5.3 |
| DOXERCALCIFEROL | -9.5 | -6.7 |
| DOXORUBICIN | -9.3 | -6.0 |
| DOXYCYCLINE | -8.4 | -6.5 |
| DOXYLAMINE | -6.5 | -4.7 |
| DROMOSTANOLONE PROPIONATE | -8.7 | -6.6 |
| DRONABINOL | -8.2 | -5.8 |
| DRONEDARONE | -8.0 | -5.4 |
| DROPERIDOL | -9.6 | -6.8 |
| DROSPIRENONE | -10.6 | -6.9 |
| DULOXETINE | -6.8 | -6.6 |
| DUTASTERIDE | -10.2 | -7.1 |
| DUVELISIB | -9.3 | -6.7 |
| DYCLONINE | -6.9 | -5.4 |
| DYDROGESTERONE | -8.6 | -6.4 |
| DYPHYLLINE | -6.6 | -5.4 |
| ECHOTHIOPHATE | -4.6 | -3.8 |
| ECONAZOLE | -7.9 | -6.0 |
| EDARAVONE | -6.4 | -5.9 |
| EDROPHONIUM | -5.9 | -4.1 |
| EFAVIRENZ | -7.2 | -5.2 |
| EFLORNITHINE | -4.9 | -4.5 |
| ELAGOLIX | -8.7 | -5.9 |
| ELETRIPTAN | -8.0 | -6.8 |
| EMEDASTINE | -7.0 | -5.3 |
| EMTRICITABINE | -6.4 | -4.9 |
| ENALAPRIL | -7.7 | -6.0 |
| ENALAPRILAT | -7.7 | -6.5 |
| ENASIDENIB | -9.1 | -7.2 |
| ENCORAFENIB | -8.4 | -7.1 |
| ENFLURANE | -4.9 | -4.7 |
| ENOXACIN | -8.0 | -5.6 |
| ENTACAPONE | -7.2 | -5.9 |
| ENTECAVIR | -7.3 | -6.0 |
| EPINASTINE | -8.8 | -5.9 |
| EPINEPHRINE | -6.0 | -5.2 |
| EPIRUBICIN | -8.4 | -5.7 |
| EPOPROSTENOL | -7.5 | -5.3 |
| EPROSARTAN | -7.5 | -5.7 |
| EPTIFIBATIDE | -7.4 | -6.6 |
| EQUILIN | -8.5 | -7.0 |
| ERAVACYCLINE | -8.7 | -6.7 |
| ERDAFITINIB | -8.1 | -5.7 |
| ERGOCALCIFEROL | -9.6 | -6.4 |
| ERIBULIN | -10.3 | -7.8 |
| ERLOTINIB | -7.9 | -6.4 |
| ERTAPENEM | -9.1 | -6.6 |
| ERTUGLIFLOZIN | -8.2 | -6.7 |
| ERYTHROMYCIN | -8.4 | -5.2 |
| ERYTHROMYCIN ESTOLATE | -8.5 | -5.1 |
| ERYTHROMYCIN ETHYLSUCCINATE | -8.9 | -5.1 |
| ESCITALOPRAM | -7.5 | -5.5 |
| ESKETAMINE | -6.3 | -5.9 |
| ESMOLOL | -6.5 | -5.1 |
| ESTAZOLAM | -8.0 | -5.8 |
| ESTRADIOL | -8.2 | -6.3 |
| ESTRADIOL CYPIONATE | -9.6 | -7.3 |
| ESTRADIOL VALERATE | -8.6 | -6.0 |
| ESTRAMUSTINE PHOSPHATE | -7.9 | -6.2 |
| ESTRONE | -8.5 | -6.1 |
| ESTROPIPATE | -9.1 | -6.5 |
| ESZOPICLONE | -8.3 | -5.9 |
| ETELCALCETIDE | -8.0 | -5.7 |
| ETHAMBUTOL | -4.8 | -3.8 |
| ETHCHLORVYNOL | -5.0 | -4.7 |
| ETHINAMATE | -6.4 | -5.4 |
| ETHINYL ESTRADIOL | -8.6 | -6.5 |
| ETHIONAMIDE | -5.4 | -5.2 |
| ETHOPROPAZINE | -7.0 | -4.8 |
| ETHOSUXIMIDE | -5.7 | -4.8 |
| ETHOTOIN | -6.5 | -5.8 |
| ETHOXZOLAMIDE | -6.4 | -5.4 |
| ETHYLESTRENOL | -7.8 | -6.2 |
| ETHYNODIOL DIACETATE | -8.2 | -6.4 |
| ETIDOCAINE | -6.4 | -4.8 |
| ETIDRONATE | -5.4 | -4.2 |
| ETODOLAC | -7.1 | -5.4 |
| ETOMIDATE | -6.9 | -5.7 |
| ETONOGESTREL | -8.5 | -5.7 |
| ETOPOSIDE | -9.6 | -6.4 |
| ETOPOSIDE PHOSPHATE | -8.8 | -6.1 |
| ETRETINATE | -7.7 | -5.9 |
| EXEMESTANE | -8.5 | -5.9 |
| EZETIMIBE | -8.2 | -6.0 |
| EZOGABINE | -7.5 | -6.0 |
| FAMCICLOVIR | -7.1 | -5.0 |
| FAMOTIDINE | -6.5 | -5.8 |
| FELBAMATE | -6.7 | -6.3 |
| FELODIPINE | -6.9 | -4.8 |
| FENOFIBRATE | -8.1 | -6.2 |
| FENOFIBRIC ACID | -7.9 | -6.8 |
| FENOLDOPAM | -7.9 | -5.7 |
| FENOPROFEN | -7.3 | -5.9 |
| FENTANYL | -8.0 | -5.8 |
| FESOTERODINE | -7.2 | -5.4 |
| FEXOFENADINE | -9.2 | -6.2 |
| FINASTERIDE | -8.5 | -6.1 |
| FINGOLIMOD | -6.7 | -4.6 |
| FLAVOXATE | -8.9 | -6.7 |
| FLECAINIDE | -8.1 | -6.1 |
| FLOXURIDINE | -6.7 | -5.1 |
| FLUCONAZOLE | -7.6 | -6.3 |
| FLUCYTOSINE | -5.1 | -4.6 |
| FLUDARABINE PHOSPHATE | -7.2 | -5.7 |
| FLUDROCORTISONE ACETATE | -8.7 | -7.0 |
| FLUMAZENIL | -7.9 | -5.6 |
| FLUNISOLIDE | -9.6 | -6.3 |
| FLUOCINONIDE | -9.6 | -7.5 |
| FLUOROMETHOLONE | -8.9 | -6.6 |
| FLUOROURACIL | -5.1 | -4.5 |
| FLUOXETINE | -7.5 | -6.7 |
| FLUOXYMESTERONE | -8.9 | -6.7 |
| FLUPHENAZINE | -8.0 | -5.9 |
| FLUPREDNISOLONE | -9.0 | -6.4 |
| FLURAZEPAM | -7.9 | -5.4 |
| FLURBIPROFEN | -7.8 | -5.6 |
| FLUTAMIDE | -7.1 | -5.7 |
| FLUTICASONE FUROATE | -10.0 | -6.3 |
| FLUTICASONE PROPIONATE | -8.9 | -6.4 |
| FLUVASTATIN | -7.9 | -5.8 |
| FLUVOXAMINE | -6.7 | -4.9 |
| FOLIC_ACID | -8.9 | -6.9 |
| FOMEPIZOLE | -4.0 | -3.8 |
| FONDAPARINUX | -5.8 | -4.4 |
| FORMOTEROL | -7.5 | -5.7 |
| FOSAPREPITANT | -9.3 | -7.0 |
| FOSCARNET | -4.5 | -3.6 |
| FOSFOMYCIN | -4.7 | -3.5 |
| FOSINOPRIL | -7.2 | -6.2 |
| FOSNETUPITANT | -9.9 | -6.2 |
| FOSPHENYTOIN | -8.7 | -6.6 |
| FOSTAMATINIB | -9.1 | -7.1 |
| FROVATRIPTAN | -7.5 | -5.7 |
| FULVESTRANT | -8.8 | -5.5 |
| FURAZOLIDONE | -6.7 | -5.7 |
| FUROSEMIDE | -7.6 | -5.6 |
| GABAPENTIN | -5.5 | -5.8 |
| GABAPENTIN ENACARBIL | -7.2 | -5.9 |
| GALANTAMINE | -7.7 | -5.0 |
| GALANTHAMINE | -7.8 | -5.7 |
| GALLAMINE | -5.6 | -4.6 |
| GANCICLOVIR | -6.9 | -5.2 |
| GANIRELIX | -6.2 | -6.4 |
| GATIFLOXACIN | -8.1 | -5.5 |
| GEFITINIB | -8.4 | -6.2 |
| GEMCITABINE | -6.9 | -6.0 |
| GEMFIBROZIL | -6.8 | -5.3 |
| GEMIFLOXACIN | -8.3 | -6.3 |
| GENTAMICIN | -7.3 | -5.9 |
| GENTIAN VIOLET | -6.9 | -6.1 |
| GILTERITINIB | -8.8 | -6.9 |
| GLASDEGIB | -8.6 | -6.8 |
| GLECAPREVIR | -11.5 | -6.7 |
| GLIMEPIRIDE | -10.1 | -7.5 |
| GLIPIZIDE | -10.1 | -7.1 |
| GLUCONOLACTONE | -5.6 | -5.1 |
| GLUTAMINE | -5.0 | -5.3 |
| GLUTATHIONE DISULFIDE | -6.9 | -6.2 |
| GLUTETHIMIDE | -7.2 | -5.1 |
| GLYCINE | -3.4 | -5.8 |
| GLYCOPYRROLATE | -7.7 | -6.0 |
| GLYCOPYRRONIUM | -7.7 | -5.6 |
| GOSERELIN | -6.6 | -5.5 |
| GRAMICIDIN | -5.3 | -5.2 |
| GRANISETRON | -8.0 | -6.5 |
| GREPAFLOXACIN | -7.8 | -6.0 |
| GRISEOFULVIN | -7.3 | -5.4 |
| GUAIFENESIN | -5.8 | -4.6 |
| GUANABENZ | -6.6 | -5.3 |
| GUANADREL | -6.8 | -6.0 |
| GUANETHIDINE | -6.3 | -5.4 |
| GUANFACINE | -6.9 | -5.7 |
| GUANIDINE | -3.2 | -2.7 |
| HALAZEPAM | -8.1 | -5.8 |
| HALCINONIDE | -10.4 | -7.0 |
| HALOFANTRINE | -8.0 | -4.7 |
| HALOPERIDOL | -8.9 | -6.8 |
| HALOPROGIN | -5.6 | -4.1 |
| HALOTHANE | -4.1 | -4.0 |
| HETACILLIN | -8.3 | -5.4 |
| HEXACHLOROPHENE | -7.8 | -5.1 |
| HEXOCYCLIUM | -7.9 | -5.5 |
| HEXYLCAINE | -7.0 | -5.3 |
| HISTAMINE | -4.3 | -4.1 |
| HISTRELIN | -7.5 | -5.4 |
| HOMATROPINE METHYLBROMIDE | -7.4 | -5.9 |
| HYDRALAZINE | -6.9 | -5.9 |
| HYDROCHLOROTHIAZIDE | -7.6 | -5.7 |
| HYDROCODONE | -7.9 | -5.7 |
| HYDROCORTAMATE | -8.3 | -6.4 |
| HYDROCORTISONE | -9.0 | -5.6 |
| HYDROCORTISONE ACETATE | -8.9 | -6.6 |
| HYDROCORTISONE BUTYRATE | -9.0 | -6.1 |
| HYDROCORTISONE CYPIONATE | -9.7 | -7.5 |
| HYDROCORTISONE PROBUTATE | -8.4 | -5.5 |
| HYDROCORTISONE VALERATE | -8.9 | -6.4 |
| HYDROFLUMETHIAZIDE | -7.9 | -5.8 |
| HYDROMORPHONE | -7.7 | -5.7 |
| HYDROXYAMPHETAMINE | -5.8 | -5.3 |
| HYDROXYCHLOROQUINE | -6.4 | -4.9 |
| HYDROXYPROGESTERONE CAPROATE | -9.3 | -5.5 |
| HYDROXYSTILBAMIDINE | -7.8 | -5.6 |
| HYDROXYUREA | -3.9 | -3.8 |
| HYDROXYZINE | -6.7 | -5.6 |
| IBANDRONATE | -6.0 | -4.6 |
| IBUPROFEN | -6.6 | -6.2 |
| IBUTILIDE | -6.9 | -5.1 |
| ICATIBANT | -7.3 | -4.9 |
| IDARUBICIN | -10.5 | -6.5 |
| IDOXURIDINE | -7.0 | -5.4 |
| IFOSFAMIDE | -5.2 | -4.4 |
| IMATINIB | -9.0 | -7.9 |
| IMIPENEM | -7.3 | -4.8 |
| IMIPRAMINE | -7.3 | -5.4 |
| IMIQUIMOD | -7.2 | -7.2 |
| IMN | -8.6 | -5.8 |
| INDACATEROL | -8.8 | -6.8 |
| INDAPAMIDE | -8.6 | -6.9 |
| INDECAINIDE | -7.3 | -4.9 |
| INDINAVIR | -9.6 | -7.1 |
| INDOMETHACIN | -8.3 | -5.6 |
| INGENOL MEBUTATE | -8.6 | -6.1 |
| IPRATROPIUM | -7.2 | -5.4 |
| IRBESARTAN | -9.0 | -6.1 |
| IRINOTECAN | -10.5 | -8.1 |
| ISOCARBOXAZID | -7.3 | -5.8 |
| ISOETHARINE | -6.6 | -4.9 |
| ISOFLURANE | -4.8 | -4.7 |
| ISOFLUROPHATE | -4.9 | -3.7 |
| ISONIAZID | -6.0 | -5.2 |
| ISOPROPAMIDE | -6.6 | -5.2 |
| ISOPROTERENOL | -6.4 | -5.1 |
| ISOSORBIDE | -5.0 | -4.2 |
| ISOSORBIDE DINITRATE | -6.6 | -4.8 |
| ISOSORBIDE MONONITRATE | -5.9 | -4.7 |
| ISOTRETINOIN | -7.7 | -6.2 |
| ISRADIPINE | -7.1 | -5.1 |
| ITRACONAZOLE | -7.5 | -7.5 |
| IVACAFTOR | -8.2 | -7.0 |
| IVOSIDENIB | -8.6 | -5.7 |
| IXABEPILONE | -8.9 | -6.1 |
| JFD00715 | -7.5 | -5.7 |
| KANAMYCIN | -7.3 | -5.8 |
| KETAMINE | -6.7 | -4.7 |
| KETOCONAZOLE | -8.8 | -6.9 |
| KETOPROFEN | -7.9 | -7.1 |
| KETOROLAC | -7.6 | -5.9 |
| KETOTIFEN | -8.8 | -5.7 |
| LABETALOL | -7.8 | -6.3 |
| LACOSAMIDE | -6.5 | -5.2 |
| LACTIC ACID | -4.0 | -3.5 |
| LAMIVUDINE | -6.4 | -5.3 |
| LAMOTRIGINE | -7.2 | -7.0 |
| LANREOTIDE | -5.9 | -4.4 |
| LANSOPRAZOLE | -8.5 | -5.9 |
| LAPATINIB | -10.2 | -7.3 |
| LAROTRECTINIB | -9.8 | -6.5 |
| LATANOPROSTENE BUNOD | -7.3 | -5.1 |
| LEFLUNOMIDE | -7.6 | -6.4 |
| LENALIDOMIDE | -7.5 | -5.7 |
| LETERMOVIR | -9.0 | -6.9 |
| LETROZOLE | -8.3 | -5.7 |
| LEUCOVORIN | -8.4 | -6.7 |
| LEVALBUTEROL | -6.7 | -5.9 |
| LEVALLORPHAN | -7.8 | -5.4 |
| LEVAMISOLE | -6.1 | -5.8 |
| LEVETIRACETAM | -5.4 | -4.3 |
| LEVOBETAXOLOL | -6.7 | -5.1 |
| LEVOBUNOLOL | -7.3 | -5.3 |
| LEVOBUPIVACAINE | -7.1 | -5.7 |
| LEVOCABASTINE | -8.4 | -7.9 |
| LEVOCARNITINE | -4.6 | -4.2 |
| LEVOCETIRIZINE | -8.0 | -5.9 |
| LEVODOPA | -6.6 | -6.1 |
| LEVOFLOXACIN | -8.3 | -6.0 |
| LEVOMEFOLATE | -8.9 | -6.9 |
| LEVOMEPROMAZINE | -7.3 | -4.9 |
| LEVOMETHADYL ACETATE | -6.6 | -4.7 |
| LEVONORDEFRIN | -6.2 | -5.8 |
| LEVONORGESTREL | -8.5 | -6.2 |
| LEVOPROPOXYPHENE | -6.8 | -4.8 |
| LEVORPHANOL | -7.9 | -5.4 |
| LEVOTHYROXINE | -6.5 | -4.6 |
| L-GLUTAMINE | -5.1 | -4.7 |
| L-HISTAMINE | -4.3 | -7.3 |
| LIDOCAINE | -6.2 | -6.5 |
| LIFITEGRAST | -10.4 | -5.2 |
| LINAGLIPTIN | -9.8 | -6.6 |
| LINCOMYCIN | -6.7 | -5.3 |
| LINEZOLID | -8.0 | -4.8 |
| LIOTHYRONINE | -7.4 | -5.3 |
| LISDEXAMFETAMINE | -6.4 | -4.6 |
| LISINOPRIL | -7.2 | -4.8 |
| LOFEXIDINE | -6.5 | -5.9 |
| LOMEFLOXACIN | -8.2 | -6.1 |
| LOMUSTINE | -6.3 | -5.3 |
| LOPERAMIDE | -8.4 | -6.3 |
| LOPINAVIR | -8.7 | -5.9 |
| LORACARBEF | -8.2 | -6.0 |
| LORATADINE | -8.0 | -6.3 |
| LORAZEPAM | -7.8 | -5.9 |
| LORLATINIB | -8.9 | -6.2 |
| LOSARTAN | -8.3 | -6.4 |
| LOTEPREDNOL ETABONATE | -9.0 | -5.8 |
| LOVASTATIN | -8.2 | -6.2 |
| LOXAPINE | -8.5 | -5.9 |
| LUBIPROSTONE | -7.4 | -5.7 |
| LUMEFANTRINE | -7.6 | -4.7 |
| LURASIDONE | -10.2 | -7.9 |
| LUSUTROMBOPAG | -8.4 | -6.5 |
| MACIMORELIN | -9.0 | -6.4 |
| MAFENIDE | -6.6 | -4.9 |
| MAPROTILINE | -7.7 | -6.1 |
| MARAVIROC | -9.1 | -6.3 |
| MASOPROCOL | -7.3 | -6.0 |
| MAYTANSINE | -8.7 | -5.4 |
| MAZINDOL | -8.6 | -5.9 |
| MEBENDAZOLE | -8.1 | -6.6 |
| MEBUTAMATE | -5.5 | -5.5 |
| MECAMYLAMINE | -5.8 | -4.5 |
| MECHLORETHAMINE | -3.6 | -3.1 |
| MECLIZINE | -9.4 | -6.6 |
| MECLOCYCLINE | -8.6 | -6.4 |
| MECLOFENAMATE | -7.8 | -6.3 |
| MEDROXYPROGESTERONE ACETATE | -8.4 | -6.1 |
| MEDRYSONE | -8.7 | -6.6 |
| MEFENAMIC ACID | -7.9 | -5.5 |
| MEFLOQUINE | -8.5 | -6.1 |
| MEGESTROL ACETATE | -8.6 | -6.2 |
| MELOXICAM | -8.7 | -6.1 |
| MELPHALAN | -6.2 | -4.8 |
| MEMANTINE | -6.3 | -5.2 |
| MENADIOL | -6.5 | -5.6 |
| MENADIONE | -6.8 | -5.4 |
| MENAQUINONE | -9.2 | -4.9 |
| MENTHOL | -5.8 | -5.3 |
| MEPENZOLATE | -7.8 | -5.8 |
| MEPERIDINE | -6.8 | -4.9 |
| MEPHENTERMINE | -5.6 | -5.0 |
| MEPHENYTOIN | -6.9 | -4.9 |
| MEPIVACAINE | -7.1 | -5.3 |
| MEPREDNISONE | -8.7 | -6.5 |
| MEPROBAMATE | -5.6 | -5.2 |
| MEQUINOL | -5.1 | -4.7 |
| MERCAPTOPURINE | -5.1 | -4.9 |
| MEROPENEM | -8.0 | -5.9 |
| MESALAMINE | -5.7 | -5.3 |
| MESNA | -4.2 | -3.4 |
| MESORIDAZINE | -8.3 | -5.7 |
| MESTRANOL | -8.3 | -6.6 |
| METAPROTERENOL | -5.8 | -6.0 |
| METARAMINOL | -6.0 | -6.0 |
| METAXALONE | -6.9 | -5.5 |
| METFORMIN | -4.7 | -4.2 |
| METHACHOLINE | -4.8 | -3.8 |
| METHACYCLINE | -8.3 | -6.7 |
| METHADONE | -6.6 | -5.8 |
| METHAMPHETAMINE | -5.3 | -5.3 |
| METHARBITAL | -6.1 | -4.7 |
| METHAZOLAMIDE | -6.4 | -4.9 |
| METHDILAZINE | -8.0 | -5.4 |
| METHICILLIN | -7.2 | -5.5 |
| METHIMAZOLE | -3.8 | -3.6 |
| METHIXENE | -8.5 | -5.8 |
| METHOCARBAMOL | -6.2 | -5.7 |
| METHOHEXITAL | -7.1 | -4.7 |
| METHOTREXATE | -8.6 | -6.5 |
| METHOXAMINE | -5.8 | -5.6 |
| METHOXSALEN | -7.1 | -5.1 |
| METHOXYFLURANE | -4.4 | -4.0 |
| METHSCOPOLAMINE | -7.6 | -5.9 |
| METHSUXIMIDE | -6.8 | -6.4 |
| METHYCLOTHIAZIDE | -6.9 | -5.9 |
| METHYL AMINOLEVULINATE | -4.7 | -4.2 |
| METHYL SALICYLATE | -5.3 | -4.8 |
| METHYLDOPA | -6.8 | -4.8 |
| METHYLDOPATE | -6.3 | -5.9 |
| METHYLERGONOVINE | -8.2 | -6.4 |
| METHYLNALTREXONE | -7.9 | -6.0 |
| METHYLPHENIDATE | -6.9 | -6.3 |
| METHYLPREDNISOLONE | -8.8 | -6.1 |
| METHYLPREDNISOLONE ACETATE | -8.3 | -6.9 |
| METHYLPREDNISOLONE SUCCINATE | -8.3 | -6.8 |
| METHYLTESTOSTERONE | -8.8 | -6.4 |
| METHYSERGIDE | -7.7 | -6.1 |
| METIPRANOLOL | -6.7 | -5.2 |
| METOCLOPRAMIDE | -6.3 | -4.9 |
| METOCURINE | -10.2 | -6.7 |
| METOLAZONE | -8.7 | -6.6 |
| METOPROLOL | -6.2 | -5.1 |
| METRONIDAZOLE | -5.6 | -4.7 |
| METYRAPONE | -6.6 | -6.0 |
| METYROSINE | -6.5 | -5.8 |
| MEXILETINE | -5.8 | -4.9 |
| MEZLOCILLIN | -9.0 | -7.1 |
| MICONAZOLE | -7.2 | -5.5 |
| MIDAZOLAM | -8.6 | -6.0 |
| MIDODRINE | -6.3 | -4.9 |
| NABILONE | -7.8 | -5.4 |
| NABUMETONE | -6.9 | -5.9 |
| NADOLOL | -7.3 | -6.0 |
| NAFARELIN | -9.7 | -6.1 |
| NAFCILLIN | -8.4 | -5.9 |
| NAFTIFINE | -8.2 | -5.9 |
| NALBUPHINE | -7.8 | -6.1 |
| NALDEMEDINE | -10.2 | -7.2 |
| NALIDIXIC_ACID | -6.6 | -5.1 |
| NALMEFENE | -8.1 | -5.6 |
| NALOXONE | -7.4 | -5.4 |
| NALTREXONE | -7.9 | -5.5 |
| NANDROLONE | -8.5 | -5.8 |
| NANDROLONE DECANOATE | -8.4 | -6.2 |
| NANDROLONE PHENPROPIONATE | -9.1 | -7.2 |
| NAPHAZOLINE | -6.9 | -5.4 |
| NAPROXEN | -7.4 | -6.8 |
| NARATRIPTAN | -7.8 | -6.3 |
| NATAMYCIN | -7.4 | -6.2 |
| NATEGLINIDE | -7.9 | -6.1 |
| NEBIVOLOL | -9.4 | -6.9 |
| NEDOCROMIL | -7.5 | -5.5 |
| NEFAZODONE | -8.7 | -6.1 |
| NELARABINE | -6.7 | -5.8 |
| NELFINAVIR | -9.0 | -6.2 |
| NEOMYCIN | -7.0 | -5.3 |
| NERATINIB | -9.0 | -6.6 |
| NETARSUDIL | -9.9 | -7.3 |
| NEVIRAPINE | -7.8 | -5.4 |
| NIACIN | -5.2 | -4.7 |
| NICARDIPINE | -8.1 | -5.8 |
| NICLOSAMIDE | -7.9 | -6.2 |
| NICOTINE | -5.4 | -5.1 |
| NIFEDIPINE | -7.2 | -4.9 |
| NILOTINIB | -9.8 | -7.7 |
| NILUTAMIDE | -7.7 | -5.9 |
| NIMODIPINE | -7.5 | -4.8 |
| NIRAPARIB | -8.6 | -6.6 |
| NITAZOXANIDE | -7.5 | -6.1 |
| NITISINONE uff | -7.8 | -5.9 |
| NITROFURANTOIN | -7.2 | -5.8 |
| NITROFURAZONE | -6.5 | -5.5 |
| NITROGLYCERIN | -5.7 | -4.6 |
| NIZATIDINE | -5.7 | -4.7 |
| NORELGESTROMIN | -8.7 | -6.3 |
| NOREPINEPHRINE | -5.9 | -5.6 |
| NORETHINDRONE | -8.5 | -6.4 |
| NORETHINDRONE ACETATE | -8.5 | -6.3 |
| NORETHYNODREL | -8.5 | -6.7 |
| NORFLOXACIN | -8.0 | -5.7 |
| NORGESTIMATE | -8.4 | -6.0 |
| NORGESTREL | -8.3 | -6.2 |
| NORTRIPTYLINE | -7.7 | -5.9 |
| NOVOBIOCIN | -9.3 | -7.2 |
| NYSTATIN | -6.8 | -4.5 |
| OCTINOXATE | -5.9 | -4.7 |
| OFLOXACIN | -7.6 | -6.2 |
| OLANZAPINE | -7.8 | -6.1 |
| OLOPATADINE | -8.0 | -6.2 |
| OLSALAZINE | -8.1 | -6.5 |
| OMADACYCLINE | -8.0 | -6.3 |
| OMEPRAZOLE | -7.5 | -6.0 |
| ONDANSETRON | -8.4 | -6.1 |
| ORLISTAT | -6.2 | -4.5 |
| ORPHENADRINE | -6.7 | -4.6 |
| OSELTAMIVIR | -7.0 | -4.7 |
| OXACILLIN | -8.5 | -6.6 |
| OXAMNIQUINE | -7.3 | -6.3 |
| OXANDROLONE | -8.5 | -6.4 |
| OXAPROZIN | -8.0 | -6.6 |
| OXAZEPAM | -7.8 | -5.7 |
| OXCARBAZEPINE | -8.1 | -6.4 |
| OXICONAZOLE | -7.3 | -6.2 |
| OXPRENOLOL | -6.1 | -4.9 |
| OXTRIPHYLLINE | -6.0 | -5.0 |
| OXYBUTYNIN | -6.9 | -4.6 |
| OXYCODONE | -7.9 | -5.7 |
| OXYMETHOLONE | -8.3 | -6.9 |
| OXYMORPHONE | -7.7 | -5.8 |
| OXYPHENBUTAZONE | -7.5 | -5.5 |
| OXYPHENCYCLIMINE | -7.9 | -5.6 |
| OXYPHENONIUM | -6.9 | -5.1 |
| OXYTETRACYCLINE | -8.2 | -6.3 |
| OZENOXACIN | -8.4 | -6.5 |
| PACLITAXEL | -10.3 | -6.9 |
| PALIPERIDONE | -10.0 | -7.5 |
| PALIPERIDONE PALMITATE | -8.7 | -6.4 |
| PALONOSETRON | -8.3 | -5.9 |
| PAMIDRONATE | -5.4 | -4.2 |
| PANCURONIUM | -8.2 | -7.0 |
| PANTOPRAZOLE | -7.9 | -5.8 |
| PANTOTHENIC ACID | -5.6 | -4.3 |
| PARAMETHADIONE | -5.5 | -4.3 |
| PARAMETHASONE ACETATE | -9.1 | -5.7 |
| PARGYLINE | -5.2 | -5.2 |
| PARICALCITOL | -8.8 | -7.0 |
| PAROMOMYCIN | -7.1 | -6.1 |
| PAROXETINE | -7.8 | -6.7 |
| PEMETREXED | -9.4 | -6.6 |
| PEMIROLAST | -7.7 | -5.8 |
| PEMOLINE | -6.3 | -6.2 |
| PENBUTOLOL | -6.8 | -5.7 |
| PENCICLOVIR | -6.6 | -5.3 |
| PENICILLAMINE | -4.3 | -3.5 |
| PENICILLIN G | -8.0 | -5.8 |
| PENICILLIN V | -7.7 | -6.0 |
| PENTAMIDINE | -6.6 | -5.8 |
| PENTAZOCINE | -8.7 | -6.0 |
| PENTOBARBITAL | -6.5 | -4.9 |
| PENTOLINIUM | -5.7 | -4.6 |
| PENTOSAN POLYSULFATE | -7.0 | -5.4 |
| PENTOSTATIN | -6.6 | -5.5 |
| PENTOXIFYLLINE | -6.6 | -5.1 |
| PERGOLIDE | -7.6 | -5.7 |
| PERPHENAZINE | -7.5 | -5.5 |
| PHENACEMIDE | -6.6 | -5.7 |
| PHENAZOPYRIDINE | -6.9 | -5.6 |
| PHENDIMETRAZINE | -5.8 | -5.6 |
| PHENELZINE | -5.9 | -5.4 |
| PHENINDIONE | -7.6 | -6.8 |
| PHENIRAMINE | -6.9 | -6.1 |
| PHENMETRAZINE | -6.2 | -5.4 |
| PHENOXYBENZAMINE | -6.0 | -4.2 |
| PHENPROCOUMON | -7.9 | -6.0 |
| PHENTERMINE | -5.5 | -5.3 |
| PHENTOLAMINE | -7.8 | -6.4 |
| PHENYLBUTAZONE | -7.6 | -5.2 |
| PHENYLBUTYRATE | -5.9 | -5.8 |
| PHENYLEPHRINE | -5.7 | -5.6 |
| PHENYLPROPANOLAMINE | -5.3 | -5.1 |
| PHENYTOIN | -8.1 | -5.9 |
| PHYTONADIONE | -8.6 | -5.3 |
| PIBRENTASVIR | -7.5 | -6.6 |
| PILOCARPINE | -5.9 | -5.3 |
| PIMOZIDE | -10.1 | -6.9 |
| PINACIDIL | -6.4 | -4.4 |
| PINDOLOL | -6.4 | -5.1 |
| PIOGLITAZONE | -8.6 | -6.3 |
| PIPECURONIUM | -7.3 | -6.3 |
| PIPERACILLIN | -9.2 | -6.4 |
| PIPERONYL BUTOXIDE | -6.4 | -5.8 |
| PIPOBROMAN | -5.3 | -4.6 |
| PIRBUTEROL | -6.5 | -5.1 |
| PIROXICAM | -8.7 | -6.4 |
| PITAVASTATIN | -8.5 | -6.1 |
| PLAZOMICIN | -7.3 | -5.6 |
| PLECANATIDE | -4.2 | -2.0 |
| PLICAMYCIN | -9.7 | -6.3 |
| PODOFILOX | -7.9 | -5.6 |
| POLYTHIAZIDE | -7.6 | -6.1 |
| POSACONAZOLE | -7.8 | -6.2 |
| PRALATREXATE | -10.0 | -7.3 |
| PRALIDOXIME | -5.3 | -6.9 |
| PRAMIPEXOLE | -5.9 | -4.9 |
| PRAMOXINE | -6.4 | -4.4 |
| PRASTERONE | -8.3 | -5.0 |
| PRASUGREL | -8.4 | -5.9 |
| PRAVASTATIN | -7.7 | -6.0 |
| PRAZEPAM | -8.1 | -6.3 |
| PRAZIQUANTEL | -8.6 | -5.5 |
| PRAZOSIN | -7.7 | -6.4 |
| PREDNICARBATE | -8.5 | -6.0 |
| PREDNISOLONE | -8.3 | -7.2 |
| PREDNISOLONE TEBUTATE | -8.8 | -6.2 |
| PREDNISONE | -8.9 | -6.3 |
| PREGABALIN | -5.2 | -6.4 |
| PRILOCAINE | -6.6 | -4.9 |
| PRIMAQUINE | -7.0 | -6.0 |
| PRIMIDONE | -6.8 | -5.1 |
| PROBENECID | -6.8 | -4.9 |
| PROBUCOL | -8.7 | -5.1 |
| PROCAINAMIDE | -6.3 | -5.1 |
| PROCAINE | -5.9 | -4.9 |
| PROCARBAZINE | -6.6 | -5.0 |
| PROCHLORPERAZINE | -7.5 | -5.2 |
| PROCYCLIDINE | -7.6 | -5.7 |
| PROGESTERONE | -8.6 | -6.3 |
| PROGUANIL | -6.9 | -6.2 |
| PROMAZINE | -7.0 | -5.1 |
| PROMETHAZINE | -7.2 | -4.7 |
| PROPAFENONE | -6.8 | -4.8 |
| PROPANTHELINE | -8.0 | -5.4 |
| PROPARACAINE | -5.6 | -6.1 |
| PROPIOMAZINE | -7.6 | -4.5 |
| PROPOFOL | -6.2 | -5.4 |
| PROPOXYPHENE | -6.7 | -5.9 |
| PROPRANOLOL | -7.2 | -4.8 |
| PROPYLTHIOURACIL | -5.5 | -5.5 |
| PROTIRELIN | -8.1 | -5.1 |
| PROTOKYLOL | -8.0 | -6.2 |
| PROTRIPTYLINE | -7.2 | -5.9 |
| PRUCALOPRIDE | -7.2 | -5.8 |
| PSEUDOEPHEDRINE | -5.7 | -5.5 |
| PTERIDINE | -8.0 | -5.2 |
| PYRAZINAMIDE | -4.9 | -4.7 |
| PYRIDOSTIGMINE | -5.6 | -4.8 |
| PYRIDOXINE | -5.3 | -5.1 |
| PYRILAMINE | -6.5 | -4.6 |
| PYRIMETHAMINE | -6.8 | -5.4 |
| PYRVINIUM | -8.0 | -7.1 |
| QUAZEPAM | -7.2 | -5.8 |
| QUETIAPINE | -7.5 | -5.8 |
| QUINAPRIL | -7.8 | -6.3 |
| QUINESTROL | -9.1 | -7.2 |
| QUINETHAZONE | -7.2 | -5.8 |
| QUINIDINE | -8.1 | -5.9 |
| QUININE | -7.4 | -6.2 |
| RABEPRAZOLE | -7.8 | -6.0 |
| RALOXIFENE | -9.4 | -6.5 |
| RAMELTEON | -7.6 | -6.0 |
| RAMIPRIL | -8.2 | -5.8 |
| RANITIDINE | -6.1 | -5.0 |
| RANOLAZINE | -8.4 | -6.3 |
| RAPACURONIUM | -5.7 | -5.6 |
| RASAGILINE | -6.1 | -4.9 |
| REGADENOSON | -7.7 | -6.4 |
| REMIFENTANIL | -6.8 | -4.8 |
| REPAGLINIDE | -8.0 | -5.6 |
| RESCINNAMINE | -9.0 | -6.5 |
| RESERPINE | -6.3 | -6.4 |
| RETAPAMULIN | -8.3 | -5.6 |
| REVEFENACIN | -10.6 | -6.9 |
| RIBAVIRIN | -6.8 | -5.4 |
| RIBOCICLIB | -8.7 | -7.3 |
| RIBOFLAVIN | -7.8 | -6.4 |
| RIBOFLAVIN PHOSPHATE | -8.3 | -6.2 |
| RIFAMYCIN | -6.3 | -6.0 |
| RIFAPENTINE | -6.4 | -6.5 |
| RIFAXIMIN | -6.6 | -5.9 |
| RILPIVIRINE | -9.2 | -6.4 |
| RILUZOLE | -6.9 | -4.8 |
| RIMANTADINE | -6.2 | -5.1 |
| RIMEXOLONE | -8.9 | -6.7 |
| RISEDRONATE | -6.5 | -5.1 |
| RISPERIDONE | -9.7 | -7.7 |
| RITODRINE | -7.7 | -4.8 |
| RITONAVIR | -9.0 | -5.8 |
| RIVAROXABAN | -9.0 | -7.1 |
| RIVASTIGMINE | -6.7 | -4.9 |
| RIZATRIPTAN | -7.0 | -5.5 |
| ROCURONIUM | -9.0 | -5.5 |
| ROFECOXIB | -8.1 | -5.8 |
| ROFLUMILAST | -7.7 | -5.7 |
| ROMIDEPSIN | -10.1 | -6.2 |
| ROPINIROLE | -6.1 | -4.8 |
| ROPIVACAINE | -7.3 | -5.3 |
| ROSIGLITAZONE | -7.7 | -5.7 |
| ROSUVASTATIN | -7.9 | -6.0 |
| ROTIGOTINE | -7.5 | -5.7 |
| RUCAPARIB | -9.4 | -6.9 |
| RUXOLITINIB | -8.5 | -6.1 |
| SAFINAMIDE | -7.8 | -6.1 |
| SALMETEROL | -7.3 | -6.0 |
| SAPROPTERIN | -7.2 | -5.4 |
| SAQUINAVIR | -9.7 | -5.3 |
| SARALASIN | -8.9 | -7.1 |
| SARECYCLINE | -8.4 | -5.9 |
| SAXAGLIPTIN | -7.8 | -5.9 |
| SCH | -7.6 | -6.1 |
| SCOPOLAMINE | -7.1 | -5.6 |
| SECNIDAZOLE | -5.9 | -4.4 |
| SECOBARBITAL | -6.7 | -4.7 |
| SEGESTERONE ACETATE | -8.7 | -6.0 |
| SELEGILINE | -5.3 | -5.4 |
| SERTACONAZOLE | -7.6 | -5.8 |
| SERTRALINE | -7.8 | -5.7 |
| SEVOFLURANE | -5.1 | -4.9 |
| SIBUTRAMINE | -6.9 | -4.6 |
| SILDENAFIL | -8.5 | -6.6 |
| SILODOSIN | -8.2 | -5.8 |
| SIMVASTATIN | -8.5 | -6.4 |
| SIPONIMOD | -9.3 | -7.1 |
| SIROLIMUS | -7.5 | -6.6 |
| SITAGLIPTIN | -8.9 | -7.1 |
| SOLIFENACIN | -8.7 | -6.2 |
| SOLRIAMFETOL | -6.1 | -5.5 |
| SORAFENIB | -9.6 | -7.7 |
| SOTALOL | -6.3 | -5.5 |
| SPARFLOXACIN | -8.1 | -6.1 |
| SPECTINOMYCIN | -7.7 | -5.4 |
| SPIRAPRIL | -7.1 | -5.7 |
| STANOZOLOL | -8.8 | -6.6 |
| STAVUDINE | -6.8 | -5.1 |
| STIRIPENTOL | -7.0 | -5.4 |
| STREPTOMYCIN | -7.6 | -5.5 |
| STREPTOZOCIN | -6.8 | -5.7 |
| SUCCIMER | -4.5 | -3.6 |
| SUCCINYLCHOLINE | -5.1 | -4.3 |
| SUFENTANIL | -7.4 | -5.2 |
| SULBACTAM | -6.6 | -4.8 |
| SULCONAZOLE | -7.5 | -5.2 |
| SULFACETAMIDE | -6.1 | -5.8 |
| SULFACYTINE | -7.3 | -5.5 |
| SULFADIAZINE | -7.0 | -5.9 |
| SULFADOXINE | -6.9 | -5.3 |
| SULFAMERAZINE | -7.4 | -5.8 |
| SULFAMETHAZINE | -7.7 | -5.9 |
| SULFAMETHIZOLE | -7.0 | -5.7 |
| SULFAMETHOXAZOLE | -7.4 | -5.4 |
| SULFANILAMIDE | -5.8 | -5.6 |
| SULFAPYRIDINE | -7.0 | -5.9 |
| SULFASALAZINE | -9.2 | -7.2 |
| SULFINPYRAZONE | -8.9 | -6.4 |
| SULFISOXAZOLE | -7.3 | -5.7 |
| SULFISOXAZOLE ACETYL | -7.3 | -5.6 |
| SULFOXONE | -7.7 | -6.5 |
| SUMATRIPTAN | -6.6 | -5.4 |
| SUNITINIB | -8.2 | -6.3 |
| SUPROFEN | -7.4 | -6.4 |
| TACRINE | -7.1 | -5.8 |
| TADALAFIL | -8.9 | -6.9 |
| TAFAMIDIS | -8.4 | -6.2 |
| TAFENOQUINE | -9.2 | -5.7 |
| TAFLUPROST | -7.3 | -5.6 |
| TALAZOPARIB | -8.7 | -6.0 |
| TAMOXIFEN | -7.9 | -5.4 |
| TAMSULOSIN | -7.7 | -5.7 |
| TAPENTADOL | -6.5 | -4.6 |
| TAZAROTENE | -7.5 | -6.1 |
| TAZOBACTAM | -7.4 | -5.2 |
| TECOVIRIMAT | -9.3 | -6.9 |
| TEGASEROD | -7.7 | -5.5 |
| TELAPREVIR | -9.5 | -6.3 |
| TELAVANCIN | -4.7 | -4.6 |
| TELBIVUDINE | -6.8 | -5.2 |
| TELMISARTAN | -10.4 | -6.7 |
| TELOTRISTAT | -10.3 | -6.8 |
| TELOTRISTAT ETHYL | -10.2 | -6.0 |
| TEMAZEPAM | -7.7 | -6.1 |
| TEMOZOLOMIDE | -6.5 | -6.6 |
| TEMSIROLIMUS | -7.4 | -6.1 |
| TENIPOSIDE | -8.8 | -6.3 |
| TENOFOVIR | -7.1 | -5.7 |
| TENOFOVIR DISOPROXIL | -6.5 | -5.8 |
| TERAZOSIN | -7.8 | -6.0 |
| TERBINAFINE | -7.7 | -5.6 |
| TERBUTALINE | -6.0 | -4.9 |
| TERCONAZOLE | -8.8 | -6.8 |
| TESTOLACTONE | -9.1 | -6.0 |
| TESTOSTERONE | -8.6 | -6.0 |
| TESTOSTERONE CYPIONATE | -9.5 | -6.7 |
| TESTOSTERONE ENANTHATE | -8.5 | -6.3 |
| TESTOSTERONE PROPIONATE | -8.5 | -6.2 |
| TETRABENAZINE | -7.5 | -5.7 |
| TETRACAINE | -6.1 | -4.9 |
| TETRACYCLINE | -8.2 | -6.3 |
| TETRAHYDROZOLINE | -7.0 | -6.6 |
| TEZACAFTOR | -9.0 | -7.3 |
| THALIDOMIDE | -7.8 | -6.4 |
| THEOPHYLLINE | -6.0 | -4.9 |
| THIABENDAZOLE | -6.5 | -5.9 |
| THIAMYLAL | -6.4 | -4.6 |
| THIETHYLPERAZINE | -7.3 | -5.4 |
| THIOGUANINE | -5.9 | -5.5 |
| THIORIDAZINE | -8.0 | -5.5 |
| THIOTHIXENE | -8.7 | -5.9 |
| THONZONIUM | -6.0 | -5.1 |
| TIAGABINE | -7.6 | -6.2 |
| TICARCILLIN | -7.3 | -6.1 |
| TICLOPIDINE | -6.7 | -5.5 |
| TIGECYCLINE | -8.3 | -6.7 |
| TILUDRONATE | -6.6 | -5.2 |
| TIMOLOL | -6.4 | -5.1 |
| TINIDAZOLE | -6.2 | -4.7 |
| TIOCONAZOLE | -6.9 | -5.4 |
| TIOPRONIN | -4.8 | -3.6 |
| TIOTROPIUM | -7.6 | -5.9 |
| TIROFIBAN | -7.4 | -5.4 |
| TIZANIDINE | -7.1 | -5.8 |
| TMC-114 | -8.5 | -5.6 |
| TOBRAMYCIN | -7.3 | -5.6 |
| TOCAINIDE | -6.3 | -5.1 |
| TOLAZAMIDE | -8.7 | -6.1 |
| TOLAZOLINE | -5.8 | -5.6 |
| TOLBUTAMIDE | -6.9 | -5.6 |
| TOLCAPONE | -8.3 | -7.3 |
| TOLMETIN | -7.6 | -5.4 |
| TOLTERODINE | -7.3 | -5.1 |
| TOLVAPTAN | -9.4 | -7.3 |
| TOPIRAMATE | -7.5 | -5.4 |
| TOPOTECAN | -8.1 | -6.1 |
| TOREMIFENE | -7.9 | -5.3 |
| TRAMADOL | -6.6 | -5.3 |
| TRANDOLAPRIL | -8.7 | -5.6 |
| TRAVOPROST | -7.9 | -6.3 |
| TRAZODONE | -8.6 | -6.7 |
| TREPROSTINIL | -8.2 | -6.0 |
| TRETINOIN | -7.9 | -6.0 |
| TRIAMCINOLONE | -9.7 | -6.6 |
| TRIAMTERENE | -7.5 | -6.5 |
| TRIAZOLAM | -7.9 | -6.1 |
| TRICHLORMETHIAZIDE | -7.4 | -5.7 |
| TRICLABENDAZOLE | -7.3 | -5.4 |
| TRICLOFOS | -5.2 | -4.3 |
| TRICLOSAN | -6.9 | -4.6 |
| TRIDIHEXETHYL | -6.9 | -6.2 |
| TRIENTINE | -4.0 | -4.1 |
| TRIFLUOPERAZINE | -7.8 | -5.9 |
| TRIFLUPROMAZINE | -7.8 | -5.1 |
| TRIFLURIDINE | -7.4 | -5.7 |
| TRIHEXYPHENIDYL | -7.9 | -5.3 |
| TRILOSTANE | -9.2 | -6.3 |
| TRIMEPRAZINE | -7.1 | -4.8 |
| TRIMETHADIONE | -5.1 | -4.3 |
| TRIMETHAPHAN | -7.8 | -5.9 |
| TRIMETHOBENZAMIDE | -7.6 | -5.3 |
| TRIMETHOPRIM | -7.0 | -5.0 |
| TRIMIPRAMINE | -7.6 | -5.5 |
| TRIPELENNAMINE | -6.6 | -5.6 |
| TRIPROLIDINE | -7.8 | -5.4 |
| TRIPTORELIN | -6.3 | -5.2 |
| TROGLITAZONE | -10.0 | -7.2 |
| TROLEANDOMYCIN | -5.8 | -5.2 |
| TROPICAMIDE | -6.8 | -5.0 |
| TROVAFLOXACIN | -9.3 | -6.4 |
| TUBOCURARINE | -8.9 | -6.8 |
| TYLOXAPOL | -6.4 | -4.5 |
| TYROSINE | -6.4 | -5.7 |
| ULIPRISTAL ACETATE | -8.9 | -6.0 |
| UNOPROSTONE ISOPROPYL | -6.6 | -5.0 |
| URACIL MUSTARD | -5.6 | -5.0 |
| URSODIOL | -8.4 | -5.9 |
| VALACYCLOVIR | -6.7 | -5.7 |
| VALBENAZINE | -7.8 | -5.7 |
| VALDECOXIB | -8.5 | -6.2 |
| VALGANCICLOVIR | -7.0 | -5.8 |
| VALPROIC ACID | -5.0 | -4.5 |
| VALRUBICIN | -9.9 | -5.8 |
| VALSARTAN | -7.7 | -5.7 |
| VANCOMYCIN | -7.7 | -5.4 |
| VANDETANIB | -8.7 | -6.9 |
| VARDENAFIL | -8.6 | -6.7 |
| VARENICLINE | -7.1 | -5.8 |
| VASOPRESSIN | -6.1 | -3.9 |
| VECURONIUM | -9.0 | -6.9 |
| VEMURAFENIB | -9.4 | -7.6 |
| VENLAFAXINE | -6.8 | -5.4 |
| VERAPAMIL | -7.6 | -5.8 |
| VIDARABINE | -6.9 | -5.0 |
| VIGABATRIN | -4.6 | -4.5 |
| VILAZODONE | -9.9 | -7.6 |
| VINBLASTINE | -8.2 | -5.7 |
| VINORELBINE | -8.5 | -4.9 |
| VIOMYCIN | -8.3 | -5.7 |
| VISMODEGIB | -9.3 | -6.9 |
| VITAMIN_A | -7.7 | -5.9 |
| VORINOSTAT | -7.1 | -5.0 |
| VOXILAPREVIR | -8.7 | -6.5 |
| WARFARIN | -8.2 | -6.3 |
| ZAFIRLUKAST | -9.6 | -7.1 |
| ZALCITABINE | -6.8 | -5.5 |
| ZALEPLON | -8.0 | -6.7 |
| ZANAMIVIR | -6.6 | -5.1 |
| ZIDOVUDINE | -6.9 | -5.5 |
| ZILEUTON | -7.0 | -5.2 |
| ZIPRASIDONE | -9.4 | -7.4 |
| ZOLMITRIPTAN | -7.6 | -6.0 |
| ZOLPIDEM | -7.7 | -6.0 |
| ZONISAMIDE | -6.6 | -5.9 |
