# Supplementary material for: Repurposing of approved drugs with potential to interact with SARS-CoV-2 receptor
Source: Biochem Biophys Rep. 2021 Mar 29;26:100982. doi: 10.1016/j.bbrep.2021.100982 (PMC8006196; doi:10.1016/j.bbrep.2021.100982)
Supplement: Supplementary file 3 [file mmc3.docx]

**Supplementary table 3:** List of anti-viral drugs that bind to ACE1 and ACE2 (Drugs listed in table 1 are shown in bold italic fonts).

| **Ligand** | **Binding energy** | | **Description (**[**Wishart DS et al., 2018**](#_ENREF_2)**;** [**Kim S et al., 2019**](#_ENREF_1)**)** |
| --- | --- | --- | --- |
|  | Human ACE1 | Human ACE2 |  |
| ABACAVIR | -8.2 | -6.3 | Abacavir is a powerful nucleoside analog reverse transcriptase inhibitor (NRTI) used to treat HIV and AIDS. |
| ACYCLOVIR | -6.7 | -5.2 | Acyclovir is a nucleotide analog antiviral used to treat herpes simplex, *Varicella zoster*, herpes zoster, herpes labialis, and acute herpetic keratitis |
| ADEFOVIR DIPIVOXIL | -7.6 | -4.8 | Adefovir dipivoxil is an acyclic nucleotide analog reverse transcriptase inhibitor (ntRTI) used for treatment of hepatitis B. It is ineffective against HIV-1. |
| AMPRENAVIR | -7.5 | -5.1 | Amprenavir is a protease inhibitor used to treat HIV infection. |
| ATAZANAVIR | -8.5 | -6.4 | Atazanavir (formerly known as BMS-232632) is an antiretroviral drug of the protease inhibitor (PI) class. Like other antiretrovirals, it is used to treat infection of human immunodeficiency virus (HIV). |
| BALOXAVIR | -9 | -6.4 | Baloxavir is an inhibitor of the influenza cap-dependent endonuclease enzyme and is used as therapy of influenza A and B. |
| ***BALOXAVIR MARBOXIL*** | -8.6 | ***-6.4*** | Baloxavirmarboxil is an antiviral drug for the treatment of influenza A and influenza B infections. |
| BICTEGRAVIR | -9.7 | -7.8 | Bictegravir is indicated in the management of HIV-1 infection. |
| BOCEPREVIR | -8.1 | -5.4 | Boceprevir is a direct acting antiviral medication used as part of combination therapy to treat chronic Hepatitis C, an infectious liver disease caused by infection with Hepatitis C Virus (HCV). |
| CIDOFOVIR | -6.4 | -5.6 | Cidofovir is an injectable antiviral medication employed in the treatment of cytomegalovirus (CMV) retinitis in patients diagnosed with AIDS. |
| DARUNAVIR | -8.8 | -5.4 | Darunavir is a protease inhibitor used with other HIV protease inhibitor drugs as well as ritonavir for the effective management of HIV-1 infection. Darunavir is being studied as a possible treatment for SARS-CoV-2, the coronavirus responsible for COVID-19, due to in vitro evidence supporting its ability to combat this infection. Clinical trials are underway and are expected to conclude in August 2020. |
| DELAVIRDINE | -8.6 | -6.6 | A potent, non-nucleoside reverse transcriptase inhibitor with activity specific for HIV-1. |
| ***DORAVIRINE*** | -8.5 | ***-6.5*** | Doravirine is an HIV-1 non-nucleoside reverse transcriptase inhibitor (NNRTI) intended to be administered in combination with other antiretroviral medicines. |
| EFAVIRENZ | -7.2 | -5.2 | Efavirenz (brand names Sustiva® and Stocrin®) is a non-nucleoside reverse transcriptase inhibitor (NNRTI) and is used as part of highly active antiretroviral therapy (HAART) for the treatment of a human immunodeficiency virus (HIV) type 1. |
| ENTECAVIR | -7.3 | -6 | Entecavir is an oral antiviral drug used in the treatment of hepatitis B infection. |
| FAMCICLOVIR | -7.1 | -5 | Famciclovir, marketed as Famvir by Novartis, is a guanine analogue used to treat herpes virus infections. |
| GANCICLOVIR | -6.9 | -5.2 | An acyclovir analog that is a potent inhibitor of the Herpesvirus family including cytomegalovirus. |
| GLECAPREVIR | -11.5 | -6.7 | Glecaprevir is a direct acting antiviral agent and Hepatitis C virus (HCV) NS3/4A protease inhibitor that targets the the viral RNA replication. |
| ***INDINAVIR*** | -9.6 | ***-7.1*** | Indinavir is an antiretroviral protease inhibitor used in the therapy and prevention of human immunodeficiency virus (HIV) infection. |
| LETERMOVIR | -9 | -6.9 | Letermovirrecieved approval from the FDA on November 8th, 2017 for use in prophylaxis of cytomegalovirus (CMV) infection in allogeneic hematopoietic stem cell transplant patients. |
| LOPINAVIR | -8.7 | -5.9 | Lopinavir is an antiretroviral protease inhibitor used in combination with other antiretrovirals in the treatment of HIV-1 infection. Lopinavir is currently under investigation in combination with ritonavir for the treatment of COVID-19 caused by SARS-CoV-2. |
| ***MARAVIROC*** | -9.1 | ***-6.3*** | A chemokine receptor antagonist drug that is designed to act against HIV by interfering with the interaction between HIV and CCR5 |
| ***NELFINAVIR*** | -9 | **-6.2** | Nelfinavir is a potent HIV-1 protease inhibitor. |
| NEVIRAPINE | -7.8 | -5.4 | A potent, non-nucleoside reverse transcriptase inhibitor (NNRTI) used in combination with nucleoside analogues for treatment of Human Immunodeficiency Virus Type 1 (HIV-1) infection and AIDS. |
| OSELTAMIVIR | -7 | -4.7 | Oseltamivir (marketed as the product TamifluⓇ), is an antiviral neuraminidase inhibitor used for the treatment and prophylaxis of infection with influenza viruses A (including pandemic H1N1) and B. |
| PENCICLOVIR | -6.6 | -5.3 | Penciclovir is a synthetic acyclic guanine derivative with antiviral activity used for the treatment of various herpes simplex virus (HSV) infections. |
| ***PIBRENTASVIR*** | ***-7.5*** | ***-6.6*** | Pibrentasvir is a direct acting antiviral agent and Hepatitis C virus (HCV) NS5A inhibitor that targets the the viral RNA replication and viron assembly. In combination with Glecaprevir, pibrentastiv is a useful therapy for patients who experienced therapeutic failure from other NS5A inhibitors. |
| REMDESIVIR | -8 | -8.1 | Remdesivir, or GS-5734, is an adenosine triphosphate analog first described in the literature in 2016 as a potential treatment for Ebola.1 In 2017, its activity against the coronavirus family of viruses was also demonstrated.2 Remdesivir is also being researched as a potential treatment to SARS-CoV-2, the coronavirus responsible for COVID-19. |
| RIBAVIRIN | -6.8 | -5.4 | Producing a broad-spectrum activity against several RNA and DNA viruses, Ribavirin is a synthetic guanosine nucleoside and antiviral agent that interferes with the synthesis of viral mRNA. It is primarily indicated for use in treating hepatitis C and viral hemorrhagic fevers. |
| RILPIVIRINE | -9.2 | -6.4 | Rilpivirine is non-nucleoside reverse transcriptase inhibitor (NNRTI) which is used for the treatment of HIV-1 infections in treatment-naive patients. |
| RITONAVIR | -9 | -5.8 | Ritonavir is an HIV protease inhibitor that interferes with the reproductive cycle of HIV. While ritonavir is not an active antiviral agent against hepatitis C virus (HCV) infection, it is added in combination therapies indicated for treatment of HCV infections as a booster. |
| SAQUINAVIR | -9.7 | -5.3 | Saquinavir is an HIV-1 protease inhibitor used in combination with ritonavir and other antiretrovirals for the treatment of human immunodeficiency virus-1 (HIV-1) infection. |
| TECOVIRIMAT | -9.3 | -6.9 | Tecovirimat is the first approved drug for smallpox |
| TELAPREVIR | -9.5 | -6.3 | Telaprevir is a direct acting antiviral medication used as part of combination therapy to treat chronic Hepatitis C, an infectious liver disease caused by infection with Hepatitis C Virus (HCV). HCV is a single-stranded RNA virus. Telaprevir is an inhibitor of NS3/4A, a serine protease enzyme, encoded by HCV genotype 1. |
| TENOFOVIR | -7.1 | -5.7 | Tenofovir has been shown to be effective against HIV, herpes simplex virus-2, and hepatitis B virus. |
| TENOFOVIR DISOPROXIL | -6.5 | -5.8 | Tenofovir is indicated in combination with other antiretroviral agents for the management of HIV-1 infection in adults and pediatric patients 2 years of age and older. It is also indicated for the treatment of chronic hepatitis B in adults and pediatric patients 12 years of age and older. |
| VALACYCLOVIR | -6.7 | -5.7 | Valaciclovir (valacyclovir), also known as *Valtrex*, is an antiviral drug that has been used to manage and treat various herpes infections for more than 2 decades. |
| VALGANCICLOVIR | -7 | -5.8 | Valganciclovir hydrochloride (Valcyte, manufactured by Roche) is an antiviral medication used to treat cytomegalovirus infections. |
| VOXILAPREVIR | -8.7 | -6.5 | Voxilaprevir is a Direct-Acting Antiviral (DAA) medication used as part of combination therapy to treat chronic Hepatitis C, an infectious liver disease caused by infection with Hepatitis C Virus (HCV). HCV is a single-stranded RNA virus. Voxilaprevir exerts its antiviral action by reversibly binding and inhibiting the NS3/4A serine protease of Hepatitis C Virus (HCV). |
| ZANAMIVIR | -6.6 | -5.1 | It is used for the prevention and treatment of influenza A and B. |
